# Diagnosis Test Accuracy of Artificial Intelligence for Endometrial Cancer: Systematic Review and Meta-Analysis

**DOI:** 10.2196/66530

**Published:** 2025-04-18

**Authors:** Longyun Wang, Zeyu Wang, Bowei Zhao, Kai Wang, Jingying Zheng, Lijing Zhao

**Affiliations:** 1 Department of Rehabilitation School of Nursing Jilin University Changchun China; 2 Department of Gynecology and Obstetrics The Second Hospital of Jilin University Changchun China

**Keywords:** artificial intelligence, endometrial cancer, diagnostic test accuracy, systematic review, meta-analysis, machine learning, deep learning

## Abstract

**Background:**

Endometrial cancer is one of the most common gynecological tumors, and early screening and diagnosis are crucial for its treatment. Research on the application of artificial intelligence (AI) in the diagnosis of endometrial cancer is increasing, but there is currently no comprehensive meta-analysis to evaluate the diagnostic accuracy of AI in screening for endometrial cancer.

**Objective:**

This paper presents a systematic review of AI-based endometrial cancer screening, which is needed to clarify its diagnostic accuracy and provide evidence for the application of AI technology in screening for endometrial cancer.

**Methods:**

A search was conducted across PubMed, Embase, Cochrane Library, Web of Science, and Scopus databases to include studies published in English, which evaluated the performance of AI in endometrial cancer screening. A total of 2 independent reviewers screened the titles and abstracts, and the quality of the selected studies was assessed using the Quality Assessment of Diagnostic Accuracy Studies—2 (QUADAS-2) tool. The certainty of the diagnostic test evidence was evaluated using the Grading of Recommendations Assessment, Development, and Evaluation (GRADE) system.

**Results:**

A total of 13 studies were included, and the hierarchical summary receiver operating characteristic model used for the meta-analysis showed that the overall sensitivity of AI-based endometrial cancer screening was 86% (95% CI 79%-90%) and specificity was 92% (95% CI 87%-95%). Subgroup analysis revealed similar results across AI type, study region, publication year, and study type, but the overall quality of evidence was low.

**Conclusions:**

AI-based endometrial cancer screening can effectively detect patients with endometrial cancer, but large-scale population studies are needed in the future to further clarify the diagnostic accuracy of AI in screening for endometrial cancer.

**Trial Registration:**

PROSPERO CRD42024519835; https://www.crd.york.ac.uk/PROSPERO/view/CRD42024519835

## Introduction

Endometrial cancer is one of the most common malignant tumors of the female reproductive system, primarily occurring in postmenopausal women [1]. Globally, the incidence of endometrial cancer is gradually increasing, mainly due to lifestyle changes, obesity, and the use of hormone replacement therapy [2,3]. According to data from the World Health Organization, there were approximately 380,000 new cases of endometrial cancer worldwide in 2020, with about 90,000 deaths [4]. The diagnosis of endometrial cancer typically requires multiple tests, such as ultrasound, biopsy, and imaging studies. These diagnostic procedures, along with subsequent treatments like surgery, radiotherapy, or chemotherapy, consume significant medical resources and incur substantial health care costs, placing a considerable burden on both patients and society [5,6].

Despite significant advances in the management of endometrial cancer, its early symptoms are often nonspecific, leading to a risk of missed or incorrect diagnosis [2]. Early diagnosis is crucial for the treatment and prognosis of endometrial cancer, as patients with early-stage endometrial cancer who undergo surgery and adjuvant therapy have a 5-year survival rate as high as 90%, while the 5-year survival rate for late-stage patients is less than 20% [7].

In traditional screening workflows, methods such as ultrasound, biopsy, computed tomography (CT), magnetic resonance imaging (MRI), and positron emission tomography are commonly used [8,9]. However, identifying abnormal imaging results can be challenging, not only being time-consuming but also potentially leading to false-positive results and overdiagnosis, which imposes additional treatment costs and psychological burdens on patients [10,11]. Currently, there is an effort to apply artificial intelligence (AI) technology to overcome these issues [12].

First, computer-aided detection systems use AI algorithms, especially deep learning models, to analyze endometrial images (such as ultrasound, MRI, and CT images) [11]. AI can act as a first reader, a second reader, or a parallel reader alongside radiologists to identify and mark abnormal areas of the endometrium, enhancing the accuracy and efficiency of imaging examinations [13]. Second, AI can be used for image preprocessing and postprocessing, including denoising, enhancement, and segmentation, helping doctors to observe and analyze the structure and lesions of the endometrium more clearly [14]. In addition, AI technology can analyze digital pathology images of endometrial biopsy samples, automatically identifying and classifying cancer cells, thereby improving the accuracy and consistency of pathological diagnosis [15]. Thus, AI-based endometrial cancer screening may open new pathways for optimizing early detection and screening workflows for endometrial cancer.

Currently, there are 10 review studies [16-25] that have explored the application of AI in endometrial cancer. However, the literature searched in these reviews is limited to a few databases [16,17,21,23], rely solely on narrative synthesis without systematic evaluation [18,19,22,24], or fail to address heterogeneity, leaving significant differences between studies undiscussed [20,25]. The research on AI applications in endometrial cancer diagnosis is progressing rapidly, and some reviews have not updated the latest developments in a timely manner. However, with the widespread adoption of AI technology, concerns have also emerged regarding its potential harms. These concerns include issues such as data bias, over-reliance on technology, privacy breaches, and the lack of algorithmic transparency, all of which may undermine the reliability and safety of AI in endometrial cancer diagnosis. Furthermore, the diagnostic accuracy of AI-based screening remains uncertain, which necessitates a systematic review to address these issues and ensure AI’s safety and effectiveness in clinical practice. This study aims to provide valuable insights and information regarding the current state of knowledge, clinical practice, policy, and future research in AI-based endometrial cancer screening. The goal is to systematically synthesize and assess the diagnostic accuracy of AI in the early detection of endometrial cancer.

## Methods

### Overview

This systematic review was performed in accordance with the PRISMA-DTA (Preferred Reporting Items for Systematic Reviews and Meta-Analyses of Diagnostic Test Accuracy Studies) guidelines (see Table S1 in Multimedia Appendix 1) [26]. This review was registered in the International Prospective Register of Systematic Reviews (PROSPERO; CRD42024519835). This study was conducted in full adherence to the registered protocol with no deviations.

### Eligibility Criteria

We included studies involving adults who were screened for endometrial cancer either through scheduled screening programs or as part of a broader health checkup (chance screening). For studies that specified the screening type, we recorded whether it was voluntary or systematic. However, most studies did not differentiate between these two types in relation to AI diagnostic performance, so we did not conduct subgroup analyses based on screening type and included all eligible studies. The index test includes an AI algorithm for diagnosing endometrial cancer to detect it early. We included reference criteria that had been elucidated by the study, including medical professional interpretation of hospital images, histopathological confirmation of tissue biopsy, surgical resection, hysteroscopy, or follow-up. The purpose of this study was to explore the diagnosis of endometrial cancer by AI, and studies involving other types of endometrial diseases were excluded. We included all diagnostic accuracy studies in English, regardless of the year of publication, excluding editorials, abstracts, and reviews. Included studies must provide diagnostic accuracy estimates, two-by-two data (true-negative [TN], true-positive ([TP], false-negative [FN], and false-positive [FP]), or other information sufficient to calculate the estimates.

### Search Strategy

Computer retrieval is the main retrieval method. We conducted a preliminary search in 5 databases: PubMed, Embase, Cochrane Library, Web of Science, and Scopus, and the initial keywords were “endometrial cancer” and “artificial intelligence.” In addition to computer-based retrieval, we also used supplementary methods to ensure a comprehensive search. We used the search methods from previous similar studies as a reference and adjusted and optimized them according to our research needs. During the process, we first defined the research question and information requirements, then picked suitable databases and search tools. We used keywords, synonyms, and related terms and built search queries with Boolean logic operators.

Searches were performed using a combination of MeSH (Medical Subject Headings) terms and entry terms. The corresponding retrieval formula is formulated according to the characteristics of each database (see Table S2 in Multimedia Appendix 1).

### Study Selection Process

The study selection process was carried out by 2 independent reviewers (LW and ZW). Both reviewers were involved in all stages of the selection process, including screening titles, abstracts, and full-text articles. Any discrepancies between the two reviewers were resolved through discussion. If a consensus could not be reached, a third reviewer (BZ) was consulted to make the final decision. This process ensured the accuracy and consistency of the study selection. Citations and reasons for the exclusion of studies are provided in Table S3 in Multimedia Appendix 1.

### Data Extraction

A total of 2 independent review authors used data extraction tables for diagnostic experiments and extracted data in duplicate. The extracted items include characteristics of the study, that is, author, year of publication, country, and purpose; Participant characteristics, that is, the number of samples, images, or subjects; Reference criteria, thresholds, and diagnostic accuracy results, that is, TP, FP, TN, and FN, area under the curve, sensitivity, specificity, and accuracy.

### Literature Quality Evaluation

A total of 2 independent review authors used the Cochrane Collaboration’s recommended Diagnostic Accuracy Studies Quality Assessment Tool-2 (QUADAS-2) to assess the risk of bias and suitability of included articles [27]. QUADAS-2 consists of the following areas: patient selection, reference standard, index test, and flow and timing. We have tailored the QUADAS-2 tool to address AI-specific biases and more accurately assess the quality of AI diagnostic research [28]. The changes are explicitly tracked in Table S4 in Multimedia Appendix 1. If all the requirements in an individual area are assessed as “yes,” the area is considered to have a low risk of bias. If any requirement is assessed as “no,” the area is considered to have a high risk of bias. If there is insufficient information to make a judgment, the risk of bias is rated as “unclear,” in line with the QUADAS-2 guidelines. When differences in assessment arise, they are resolved through mutual discussion and consensus with a third independent evaluator.

### Diagnostic Accuracy Measures

The accuracy indexes of diagnostic measurement are mainly sensitivity and specificity; sensitivity represents the probability of detecting positive in the population judged by the gold standard as diseased (positive), and specificity represents the probability of detecting negative in the population judged by the gold standard as disease-free (negative) [29]. The more sensitive and specific a diagnostic test is, the more valuable it is.

### Synthesis of Results

Stata software was used for data analysis. Forest mapping using extracted two-by-two data with sensitivity and specificity measurements with 95% confidence contour. In addition, we used the media’s command to calculate the likelihood ratio, diagnostic odds ratio, and 95% confidence contour for meta-analysis of diagnostic studies [30]. Given the heterogeneity and unclear thresholds of endometrial cancer detection in different AI models, we used the media’s command to plot a summary receiver operating curve (SROC) curve, which includes aggregated measures of sensitivity and specificity for selected articles, area under the curve (AUC), with a 95% confidence contour [31]. To investigate potential sources of heterogeneity, we performed subgroup analyses based on subgroups of AI type, region, study type, and year of publication in the extracted information.

### Certainty of Evidence

The GRADE (Grading of Recommendations, Assessment, Development, and Evaluation) criteria were applied to assess the certainty of evidence for the entirety of diagnostic accuracy studies, focusing on 5 key domains: risk of bias, indirectness, inconsistency (significant variations in diagnostic accuracy estimates), imprecision (broad CIs), and publication bias [32,33]. Each study was independently evaluated by the reviewers. The certainty of evidence was downgraded whenever there was sufficient justification for such a decision in any of these domains.

## Results

### Study Selection

A total of 1241 records were identified by the literature search on January 1, 2024, of which 326 records were collected from PubMed, 362 from Scopus, 250 from Embase, and 39 from the Cochrane Library. In total, 264 records were collected from the Web of Science database.

After removing duplicates (n=210), the titles of 1031 records were filtered. According to the title and summary, 821 records were excluded because the content or format was not relevant. A careful reading of the full text removed 197 articles and finally included 13 articles after two researchers independently reached a consensus.

**Figure 1:**
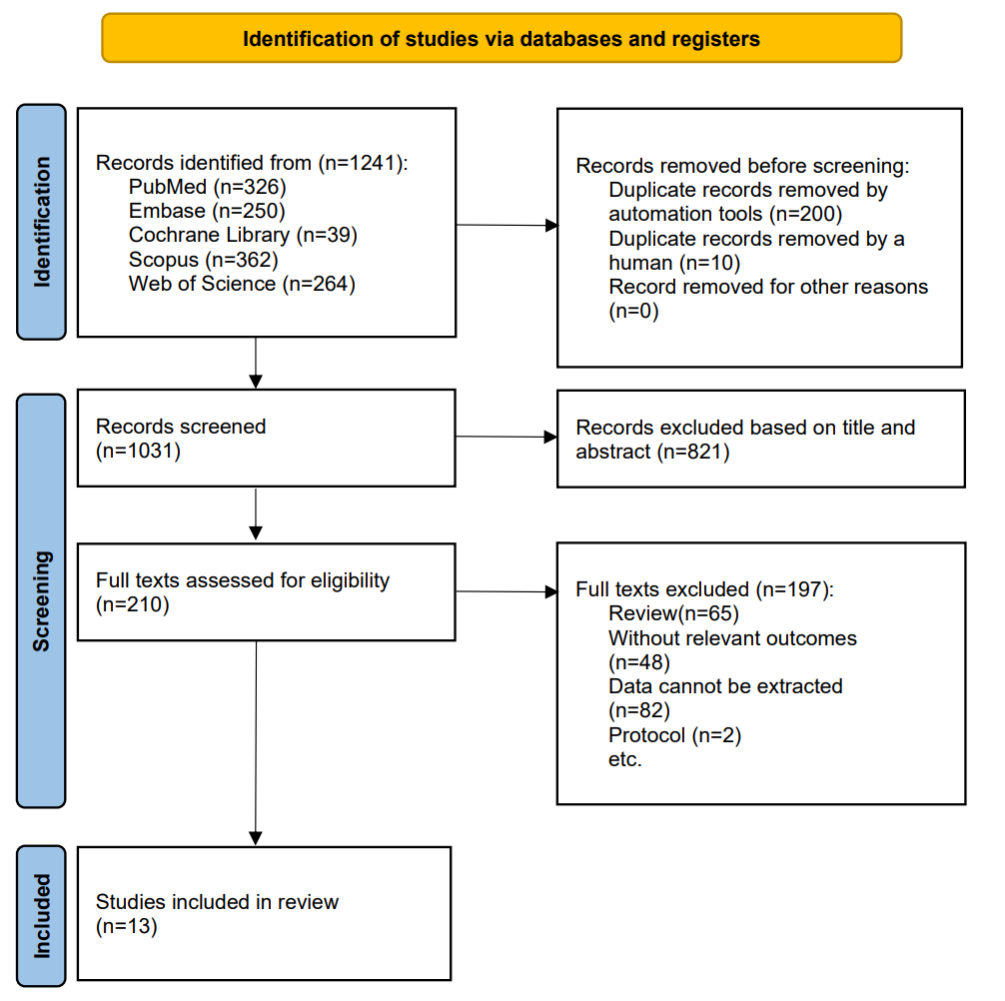
PRISMA (Preferred Reporting Items for Systematic Reviews and Meta-Analyses) 2020 flow diagram.

Figure 1 shows the study selection process and results.

### Features Included in This Study

Finally, 13 studies were included in this study, involving 6400 patients and 438,617 samples. Table 1 shows the basic characteristics of the selected articles. Additional characteristics of the included studies, including the PPV (positive predictive value), NPV (negative predictive value), Function and Objectives of the AI System, Source of Training Data, and Data-Study Population Match, are detailed in Table S5 in Multimedia Appendix 1. The included studies were published between 2017 and 2022, mainly from Asian countries (n=9), including China (n=6) and Japan (n=3), in addition to a number of non-Asian countries (n=4), including Italy (n=1), Greece (n=1), Canada (n=1), and the United States (n=1). In total, 8 of the 13 studies were retrospective, and 5 were prospective. Of the studies included, 7 used deep learning to screen for endometrial cancer, and the remaining 6 applied machine learning. There are 7 AI models included in these studies, the most common being convolutional neural network (n=6) and random forest (n=2). Of the selected articles, 2 studies used histopathology, 9 studies used expert diagnoses as reference standards, and 2 studies did not specify reference standards. Most studies (n=11) enrolled patients and collected samples from local hospitals or Cancer institutes, while others obtained patient data and samples of pathological images from public databases, such as the pathological database, the Cancer Imaging database, and the Gene Expression Omnibus database.

**Table 1:** Characteristics of included studies.

Author, year	TP^a^	FP^b^	FN^c^	TN^d^	Country	Study design	Type of AI^e^	Reference standard
Chen et al [34], 2020	12	15	6	105	China	Retrospective cohort	Deep learning (CNN^f^)	Expert readers
Chiappa et al [35], 2021	16	4	7	43	Italy	Retrospective cohort	Machine learning	Expert readers
Dongli Zhao et al [36], 2022	463	9	85	26	China	Retrospective cohort	Machine learning (RF^g^)	NR^h^
Ebrahimian et al [37], 2020	1093	295	412	1502	Canada	Retrospective cohort	Machine learning (SVM^i^)	Histopathology
Fengjun Zhao et al [38], 2022	1039	9	67	516	China	Retrospective cohort	Deep learning (CNN)	Expert readers
Hart et al [39], 2020	47	2	3	48	United States	Prospective cohort	Machine learning (RF)	Expert readers
Li et al [40], 2021	254	51	162	459	China	Retrospective cohort	Machine learning (Python)	Expert readers
Li et al [41], 2022	192	771	17	10522	China	Prospective cohort	Deep learning (CNN)	Expert readers
Makris et al [42], 2017	151	17	21	227	Greece	Prospective cohort	Machine learning (Neural network)	NR
Saida et al [43], 2022	52	3	4	7	Japan	Retrospective cohort	Deep learning (CNN)	Expert readers
Sun et al [44], 2020	46	0	13	141	China	Prospective cohort	Deep learning (CNN)	Expert readers
Takahashi et al [45], 2021	33	15	3	126	Japan	Prospective cohort	Deep learning (DNN^j^)	Histopathology
Urushibara et al [46], 2022	48	6	3	40	Japan	Retrospective cohort	Deep learning (CNN)	Expert readers

^a^TP: true-positive.

^b^FP: false-positive.

^c^FN: false-negative.

^d^TN: true-negative.

^e^AI: artificial intelligence.

^f^CNN: convolutional neural network.

^g^RF: random forest.

^h^NR: not reported.

^i^SVM: support vector machine.

^j^DNN: deep neural network.

### Risk of Bias and Applicability

**Figure 2:**
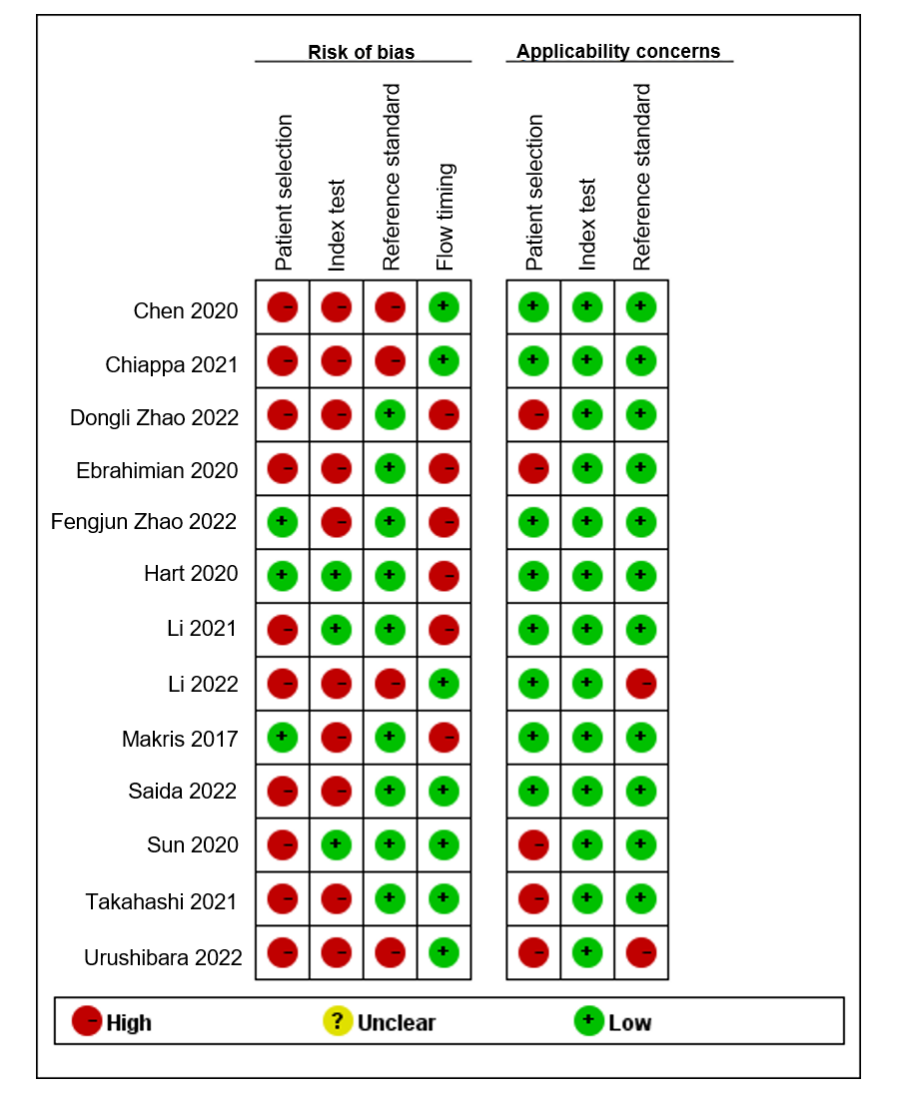
Risk of bias and applicability concerns summary [34-46].

The overall methodological quality of the study was assessed using QUADAS-2, and Figure 2 [34-46] shows the results. Of the included studies, 10/13 (77%) had a high risk of bias, mainly due to inadequate case-control design information and inappropriate exclusion criteria. Regarding the use of AI in diagnostics, a high risk of bias was found in 10/13 (77%) studies. These studies lacked a blind evaluation of the index test, and the model codes were not publicly available. In terms of process and timing, there was a high risk of bias in 6/13 (46%) studies, mainly because the time interval between the trial to be evaluated and the gold standard in some studies could not be determined, or it was not possible to determine whether all patients received only one gold standard. High applicability concerns of the evidence to patients were found in 5 (38%) studies. There are low applicability concerns regarding the evidence for the index test in all studies. There are high applicability concerns of the evidence to the reference standard in 2/13 (15%) studies.

### Results of Individual Studies

**Figure 3:**
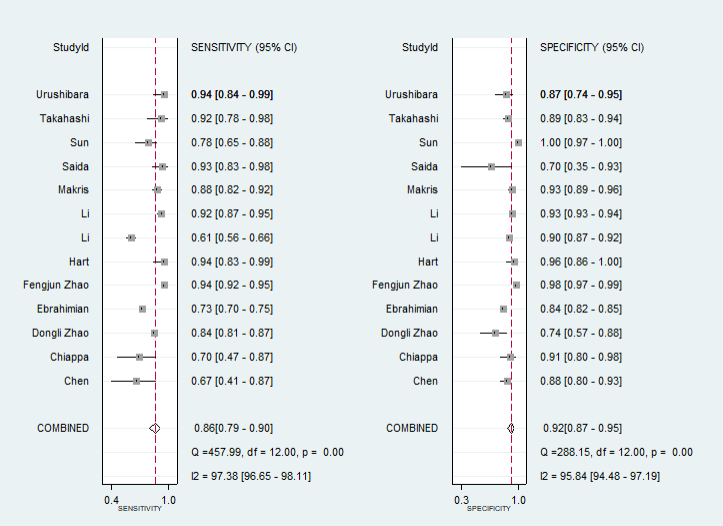
Forest plots of sensitivity and specificity in 13 included studies on using artificial intelligence (AI) for endometrial cancer screening [34-46].

Figure 3 [34-46] shows the forest plot of the sensitivity and specificity of each study. In 13 studies, the sensitivity of using AI to screen for endometrial cancer ranged from 67% to 94%, and the specificity ranged from 70% to 100%.

### Synthesis of Results

**Figure 4:**
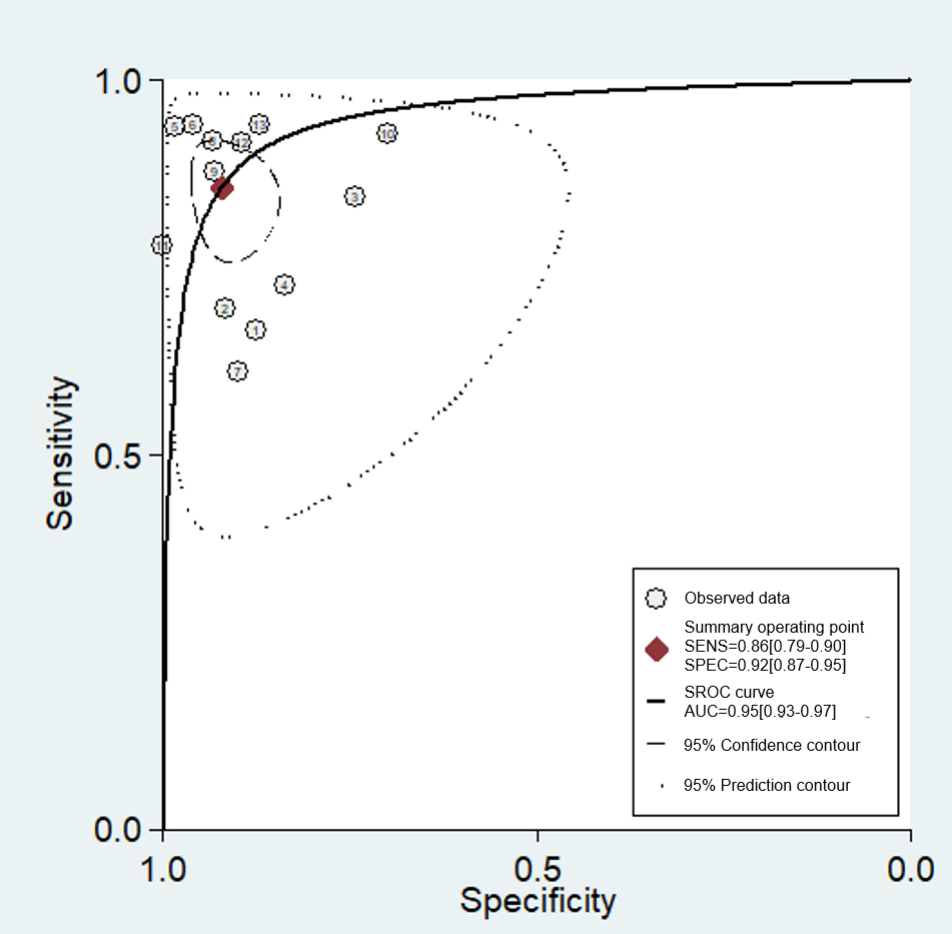
Summary receiver operating characteristic curves of included studies. SENS; sensitivity; SPEC: specificity; SROC: summary receiver operating characteristic curve; AUC: area under the curve.

Based on the sensitivity and specificity of the included studies, the SROC curve was fitted to evaluate the performance of AI systems in supporting the diagnostic process for endometrial cancer. Figure 4 shows the SROC curve, with a prediction interval of 95% and a CI of 95%, and its overall aggregated estimates are as follows: sensitivity of 86% (95% CI 79%-90%), specificity of 92% (95% CI 87%-95%), and area under the curve of 95%. Summary points are represented by a red diamond, and individual studies are represented by a circle with a number marker. The short dashed and dotted lines represent the 95% confidence and 95% prediction contour, respectively.

### Subgroup Analyses

In order to analyze the possible causes and sources of inter-study heterogeneity, we conducted a series of subgroup analyses, the detailed results of which are shown in Table 2. Specific groupings include the type of AI used in the studies (machine learning versus deep learning), region (Asian vs non-Asian), year of publication (pre-2022 and post-2022), and type of study (prospective vs retrospective), with the results of the subgroup analysis similar to those of the main analysis of AI screening for endometrial cancer.

**Table 2:** Subgroup analyses for using artificial intelligence (AI) for endometrial cancer screening.

Subgroup variables		Number of eligible studies, n	Sensitivity, % (95% CI)	Specificity, % (95% CI)
**Type of artificial intelligence**				
	Machine learning	4	81 (69-88)	89 (84-93)
	Deep learning	9	90 (84-94)	94 (85-98)
**Region**				
	Asia	9	87 (79-92)	92 (84-96)
	Non-Asia	4	84 (74-91)	91 (84-95)
**Year of publication**				
	≤2021	5	80 (70-87)	93 (87-96)
	≥2022	8	90 (86-93)	90 (72-97)
**Type of study**				
	Prospective	5	89 (84-93)	95 (89-98)
	Retrospective	8	83 (72-90)	89 (82-94)

### Certainty of Evidence

Each reviewer independently assessed the overall certainty of the evidence. The domains of risk of bias, indirectness, and consistency were downgraded due to numerous studies exhibiting a high risk of bias in patient selection, flow and timing, and reference standards, as well as significant variability in specificity. Following the GRADE approach for diagnostic tests, the certainty of evidence for the accuracy of AI-based tests in endometrial cancer screening was deemed very low for both sensitivity and specificity estimates (see Table S5 in Multimedia Appendix 1).

## Discussion

### Principal Findings

This is the first systematic review and meta-analysis to evaluate the diagnostic accuracy of AI-based screening for early-stage endometrial cancer. The analysis found that AI-based screening was able to detect 86% (95% CI 79%-90%) of endometrial cancer patients and exclude 92% (95% CI 87%-95%) of nonendometrial cancer patients. However, significant heterogeneity in study methodologies, along with a lack of consistent reporting on patient selection, processes, and timing, may introduce substantial bias. In addition, the included studies did not fully adhere to existing reporting standards for diagnostic accuracy studies, such as the Standards for Reporting Diagnostic Accuracy (STARD) [47]. This highlights the need for appropriate reporting standards to improve the quality and completeness of AI-specific diagnostic accuracy research.

While the QUADAS-2 tool provides a robust framework for assessing bias in diagnostic accuracy studies, its application to AI-based models requires contextual customization to address algorithmic and data-driven biases. Recent literature [48] has highlighted these difficulties and proposed AI-specific reporting guidelines to address biases in data curation and model transparency. In this review, we addressed these limitations by tailoring QUADAS-2 signaling questions to target AI-related confounders. Key modifications included replacing generic questions with the Patient Selection domain to detect data leakage risks and adding new signaling questions in the Index Test domain with criteria evaluating model reproducibility. We advocate for broader adoption of domain-specific tailoring of QUADAS-2 in AI diagnostic reviews, complemented by emerging tools such as PROBAST-AI [48]. Future efforts should prioritize consensus-building on standardized AI adaptations to enhance cross-study comparability.

The results of this study suggest that AI-based approaches, particularly deep learning, have the potential to support early screening for endometrial cancer by providing high sensitivity and specificity. In this study, the sensitivity of deep learning was 90% (95% CI 84%-94%) and the specificity was 94% (95% CI 85%-98%), compared with the highest sensitivity and specificity in previous studies, which were 87% (95% CI 83%-90.2%) and 92.5% (95% CI 85.1%-96.4%), respectively [11,49]. While these findings are promising, the certainty of evidence for these estimates was deemed very low, largely due to methodological limitations in the included studies. Furthermore, the pooled estimates may not fully account for challenges such as overdiagnosis, variability in clinical settings, and the impact of AI on downstream clinical decision-making. Future research should focus on addressing these gaps to provide stronger evidence for the effectiveness and safety of AI in endometrial cancer screening.

Subgroup analysis suggested that studies using deep learning reported higher sensitivity and specificity compared with those using machine learning, though this observation may be influenced by variations in study design, populations, and methodologies. Due to the limited ability of machine learning to handle complex data, it may not perform as well as deep learning when dealing with highly complex and high-dimensional data [11]. However, in cases where input imaging data is insufficient, traditional machine learning can still accurately detect endometrial cancer across different imaging modalities, retaining significant diagnostic potential comparable to deep learning in clinical applications [50,51]. Given the increasing application of AI in cancer screening and treatment, this study highlights the potential advantages of deep learning over traditional screening methods for early detection of endometrial cancer. Deep learning, particularly convolutional neural networks (CNNs), has shown promise in automatically extracting complex features from images and handling large-scale, high-dimensional datasets [10,52,53]. However, this recommendation must be interpreted cautiously, as the certainty of evidence remains low, and the potential harms of AI, such as overdiagnosis and resource implications, have not been thoroughly studied. Further research is needed to evaluate how AI-based methods compare with traditional approaches in real-world clinical settings and their overall impact on patient outcomes. However, considering the high accumulation and training costs associated with deep learning algorithms, future guidelines and policies need to be developed to adapt and adjust AI-based imaging for endometrial cancer screening tailored to the medical contexts of different countries.

In the subgroup analysis, AI-based screening demonstrated higher sensitivity in Asian endometrial cancer patients, with a correct detection rate of 87% (95% CI 79%-92%), compared with 84% (95% CI 79%-91%) in non-Asian patients, while the specificity for excluding nonendometrial cancer cases was similar between the two groups. For AI-based imaging examinations, quantitative imaging features related to tumor-associated biomarkers, such as estrogen receptor (ER) and progesterone receptor (PR), play a critical role in the early screening of endometrial cancer [54]. Existing studies suggest that there may be differences in hormone receptor expression levels across racial groups [55,56]. Given that AI must continuously track disease evolution, these racial differences in endometrial cancer biomarkers, along with the varied imaging features, may at least partially explain the superior performance of AI-based imaging in the early detection of endometrial cancer among Asian populations. However, before concluding the impact of racial differences, further evaluation is needed to assess the diagnostic accuracy differences of AI-based imaging between Asian and non-Asian populations.

Regarding the differences in diagnostic performance across studies published in different years, we found that studies conducted in 2022 and later demonstrated better diagnostic accuracy compared with those conducted in 2021 and earlier. Notably, over half of the diagnostic accuracy studies conducted in 2022 and beyond focused on the development and evaluation of deep learning performance, suggesting that the type of AI used significantly impacts diagnostic performance, with deep learning outperforming machine learning. This finding supports the use of deep learning algorithms in developing AI-based screening methods, such as computer-aided detection and diagnostic systems [11], with promising potential for clinical application. Therefore, future research should aim to further refine machine learning algorithms to enhance their performance in detecting endometrial cancer.

In addition, our subgroup analysis of the study types included revealed that prospective studies have an advantage over retrospective studies in evaluating diagnostic performance and reliability [52]. Prospective studies set clear inclusion criteria at the outset and sequentially enroll patients who meet these criteria, thus reducing selection bias. Furthermore, prospective studies collect real-time data, which more accurately reflects current clinical practice and the latest technological advancements. In contrast, retrospective studies may suffer from selection bias due to inconsistent data collection and the selective inclusion of specific cases, and their data may not reflect the most up-to-date diagnostic techniques and methods [53]. Furthermore, our analysis found that there are currently fewer prospective studies available. In the future, more prospective studies should be conducted to dynamically observe the long-term effects and reliability of AI-based diagnostic tests, providing a more comprehensive evaluation of their performance.

In this study, we strictly adhered to guidelines for diagnostic systematic reviews [57]. We applied rigorous eligibility criteria and quality assessment tools to the included studies. After a comprehensive search across multiple databases, we extracted data that could influence the diagnostic performance of AI-based endometrial cancer screening, such as TPs, FPs, FNs, TNs, AI type, study region, and publication year. Subgroup analysis was conducted to reduce the heterogeneity of the results. Despite these efforts, some inevitable limitations remain in this study. First, more than half of the included articles had a high risk of bias, particularly in patient selection. It is difficult to determine whether patient samples from some publicly accessible datasets were enrolled consecutively or randomly, which may result in the inclusion of patients with severe disease or healthy controls. This could lead to an overestimation of the pooled sensitivity and specificity of AI-based diagnostic accuracy. Second, most studies used public data sources for retrospective analyses, and only 5 prospective studies evaluated AI algorithm performance in clinical settings. This methodological limitation may have introduced bias. Third, the included studies did not consistently report test failures, limiting our ability to analyze their potential impact on diagnostic performance. Standardized reporting of test failures in future research could provide a more comprehensive evaluation of AI-based screening tools. Fourth, the lack of consistent reporting on test thresholds across studies is another limitation. Test thresholds are critical in defining positive results, and variability among AI systems may affect the comparability and synthesis of sensitivity and specificity estimates. Transparent and standardized reporting of test thresholds is necessary to improve the interpretability of future meta-analyses. Finally, excluding non–English-language studies may have led to the omission of important research on the performance of AI-based screening.

Early screening is crucial for endometrial cancer patients, as accurate identification in the early stages allows for timely management, improving patient outcomes [58]. To enhance the quality of future AI-based screening research, the following recommendations should be adopted: first, future studies should strengthen patient selection criteria to increase the reliability of the research [59]. Second, many AI algorithms are developed and evaluated on similar populations, often using subsets of the same data sources, which may lead to overly optimistic performance results compared with true external validation sets. To achieve greater generalizability and reproducibility, future research should develop AI models using large, diverse datasets from specialized hospitals, endometrial cancer screening programs, cancer research institutes, and national databases [12,60]. Third, dedicated studies should be conducted to evaluate the diagnostic performance of AI, such as comparing AI with health care professionals or assessing the combined performance of AI models and health care professionals. These studies are critical for endometrial cancer screening and will enhance the robustness of developed AI models, making them more suitable for early detection of endometrial cancer. Finally, further research is needed to explore how these AI models can be effectively integrated into endometrial cancer screening workflows and to assess their impact on patient-related clinical outcomes.

### Conclusion

The diagnostic performance of AI-based early detection of endometrial cancer is promising and holds potential clinical value. In the future, well-designed randomized controlled trials and cohort studies in large populations undergoing endometrial cancer screening are needed to compare test accuracy. These studies should assess the accuracy of AI-based screening and evaluate clinical diagnostic models that combine AI with health care professionals’ expertise.

## Appendix

Multimedia Appendix 1 PRISMA-DTA (Preferred Reporting Items for Systematic Reviews and Meta-Analyses of Diagnostic Test Accuracy Studies) checklist and other materials.

## Abbreviations

AI: artificial intelligence
AUC: area under the curve
CNN: convolutional neural network
CT: computed tomography
ER: estrogen receptor
FN: false-negative
FP: false-positive
GRADE: Grading of Recommendations Assessment, Development, and Evaluation
MeSH: Medical Subject Headings
MRI: magnetic resonance imaging
NPV: negative predictive value
PPV: positive predictive value
PR: progesterone receptor
PRISMA-DTA: Preferred Reporting Items for Systematic Reviews and Meta-Analyses of Diagnostic Test Accuracy Studies
QUADAS-2: Quality Assessment of Diagnostic Accuracy Studies—2
SROC: summary receiver operating curve
STARD: Standards for Reporting Diagnostic Accuracy
TN: true-negative
TP: true-positive

## Data Availability

All data generated or analyzed during this study are included in this published article.
