# Supplementary material for: Diagnosis Test Accuracy of Artificial Intelligence for Endometrial Cancer: Systematic Review and Meta-Analysis
Source: J Med Internet Res. 2025 Apr 18;27:e66530. doi: 10.2196/66530 (PMC12048793; doi:10.2196/66530)
Supplement: Multimedia Appendix 1 [file jmir_v27i1e66530_app1.docx]

**Table S1. Preferred Reporting Items for Systematic Reviews and Meta-analyses of Diagnostic Test Accuracy Studies (PRISMA-DTA) Checklist**

| **Section/topic** | **#** | **PRISMA-DTA Checklist Item** | **Reported on page #** |
| --- | --- | --- | --- |
| **TITLE / ABSTRACT** | | |  |
| Title | 1 | Identify the report as a systematic review (+/- meta-analysis) of diagnostic test accuracy (DTA) studies. | 1 |
| Abstract | 2 | Abstract: See PRISMA-DTA for abstracts. | 2-3 |
| **INTRODUCTION** | | |  |
| Rationale | 3 | Describe the rationale for the review in the context of what is already known. | 3-4 |
| Clinical role of index test | D1 | State the scientific and clinical background, including the intended use and clinical role of the index test, and if applicable, the rationale for minimally acceptable test accuracy (or minimum difference in accuracy for comparative design). | 3-4 |
| Objectives | 4 | Provide an explicit statement of question(s) being addressed in terms of participants, index test(s), and target condition(s). | 5 |
| **METHODS** | | |  |
| Protocol and registration | 5 | Indicate if a review protocol exists, if and where it can be accessed (e.g., Web address), and, if available, provide registration information including registration number. | 5 |
| Eligibility criteria | 6 | Specify study characteristics (participants, setting, index test(s), reference standard(s), target condition(s), and study design) and report characteristics (e.g., years considered, language, publication status) used as criteria for eligibility, giving rationale. | 5-6 |
| Information sources | 7 | Describe all information sources (e.g., databases with dates of coverage, contact with study authors to identify additional studies) in the search and date last searched. | 6 |
| Search | 8 | Present full search strategies for all electronic databases and other sources searched, including any limits used, such that they could be repeated. | 6-7 |
| Study selection | 9 | State the process for selecting studies (i.e., screening, eligibility, included in systematic review, and, if applicable, included in the meta-analysis). | 6-7 |
| Data collection process | 10 | Describe method of data extraction from reports (e.g., piloted forms, independently, in duplicate) and any processes for obtaining and confirming data from investigators. | 8 |

| **Section/topic** | **#** | **PRISMA-DTA Checklist Item** | **Reported on page #** |
| --- | --- | --- | --- |
| Definitions for data extraction | 11 | Provide definitions used in data extraction and classifications of target condition(s), index test(s), reference standard(s) and other characteristics (e.g. study design, clinical setting). | 7 |
| Risk of bias and applicability | 12 | Describe methods used for assessing risk of bias in individual studies and concerns regarding the applicability to the review question. | 7 |
| Diagnostic accuracy measures | 13 | State the principal diagnostic accuracy measure(s) reported (e.g. sensitivity, specificity) and state the unit of assessment (e.g. per-patient, per-lesion). | 7 |
| Synthesis of results | 14 | Describe methods of handling data, combining results of studies and describing variability between studies. This could include, but is not limited to: a) handling of multiple definitions of target condition. b) handling of multiple thresholds of test positivity, c) handling multiple index test readers, d) handling of indeterminate test results, e) grouping and comparing tests, f) handling of different reference standards | 8 |
| Meta-analysis | D2 | Report the statistical methods used for meta-analyses, if performed. | 8 |
| Additional analyses | 16 | Describe methods of additional analyses (e.g., sensitivity or subgroup analyses, meta-regression), if done, indicating which were pre-specified. | 8 |
| **RESULTS** | | |  |
| Study selection | 17 | Provide numbers of studies screened, assessed for eligibility, included in the review (and included in meta-analysis, if applicable) with reasons for exclusions at each stage, ideally with a flow diagram. | 8-9 |
| Study characteristics | 18 | For each included study provide citations and present key characteristics including: a) participant characteristics (presentation, prior testing), b) clinical setting, c) study design, d) target condition definition, e) index test, f) reference standard, g) sample size, h) funding sources | 9-13 |
| Risk of bias and applicability | 19 | Present evaluation of risk of bias and concerns regarding applicability for each study. | 14-15 |
| Results of individual studies | 20 | For each analysis in each study (e.g. unique combination of index test, reference standard, and positivity threshold) report 2x2 data (TP, FP, FN, TN) with estimates of diagnostic accuracy and confidence intervals, ideally with a forest or receiver operator characteristic (ROC) plot. | 15-16 |
| Synthesis of results | 21 | Describe test accuracy, including variability; if meta-analysis was done, include results and confidence intervals. | 16-17 |
| Additional analysis | 23 | Give results of additional analyses, if done (e.g., sensitivity or subgroup analyses, meta-regression; analysis of index test: failure rates, proportion of inconclusive results, adverse events). | 17-20 |
| **DISCUSSION** | | |  |
| Summary of evidence | 24 | Summarize the main findings including the strength of evidence. | 21-23 |
| Limitations | 25 | Discuss limitations from included studies (e.g. risk of bias and concerns regarding applicability) and from the review process (e.g. incomplete retrieval of identified research). | 24-25 |
| Conclusions | 26 | Provide a general interpretation of the results in the context of other evidence. Discuss implications for future research and clinical practice (e.g. the intended use and clinical role of the index test). | 26-27 |
| **FUNDING** | | |  |
| Funding | 27 | For the systematic review, describe the sources of funding and other support and the role of the funders. | 27 |

**Table S2. Specific search formula.**

| Datebase name | Search strategies: key words and how these  were combined in the search | Laster  update | Number of  studies  identified |
| --- | --- | --- | --- |
| PubMed | (((((((((((((((((((((Endometrial Neoplasms[MeSH Terms]) OR (Endometrial Neoplasm[Title/Abstract])) OR (Neoplasm, Endometrial[Title/Abstract])) OR (Neoplasms, Endometrial[Title/Abstract])) OR (Endometrial Carcinoma[Title/Abstract])) OR (Carcinoma, Endometrial[Title/Abstract])) OR (Carcinomas, Endometrial[Title/Abstract])) OR (Endometrial Carcinomas[Title/Abstract])) OR (Endometrial Cancer[Title/Abstract])) OR (Cancer, Endometrial[Title/Abstract])) OR (Cancers, Endometrial[Title/Abstract])) OR (Endometrial Cancers[Title/Abstract])) OR (Endometrium Cancer[Title/Abstract])) OR (Cancer, Endometrium[Title/Abstract])) OR (Cancers, Endometrium[Title/Abstract])) OR (Cancer of the Endometrium[Title/Abstract])) OR (Carcinoma of Endometrium[Title/Abstract])) OR (Endometrium Carcinoma[Title/Abstract])) OR (Endometrium Carcinomas[Title/Abstract])) OR (Cancer of Endometrium[Title/Abstract])) OR (Endometrium Cancers[Title/Abstract])) AND ((((((((((((((((((((Artificial Intelligence[MeSH Terms]) OR (Intelligence, Artificial[Title/Abstract])) OR (Computational Intelligence[Title/Abstract])) OR (Intelligence, Computational[Title/Abstract])) OR (Machine Intelligence[Title/Abstract])) OR (Intelligence, Machine[Title/Abstract])) OR (Computer Reasoning[Title/Abstract])) OR (Reasoning, Computer[Title/Abstract])) OR (AI (Artificial Intelligence[Title/Abstract]))) OR (Computer Vision Systems[Title/Abstract])) OR (Computer Vision System[Title/Abstract])) OR (System, Computer Vision[Title/Abstract])) OR (Systems, Computer Vision[Title/Abstract])) OR (Vision System, Computer[Title/Abstract])) OR (Vision Systems, Computer[Title/Abstract])) OR (Knowledge Acquisition (Computer[Title/Abstract]))) OR (Acquisition, Knowledge (Computer[Title/Abstract]))) OR (Knowledge Representation (Computer[Title/Abstract]))) OR (Knowledge Representations (Computer[Title/Abstract]))) OR (Representation, Knowledge (Computer[Title/Abstract]))) | 1st  January  2024 | 326 |
| Scopus | #1 ( TITLE-ABS-KEY ( endometrial AND neoplasms ) OR TITLE-ABS-KEY ( endometrial AND neoplasm ) OR TITLE-ABS-KEY ( neoplasm, AND endometrial ) OR TITLE-ABS-KEY ( neoplasms, AND endometrial ) OR TITLE-ABS-KEY ( endometrial AND carcinoma ) OR TITLE-ABS-KEY ( carcinoma, AND endometrial ) OR TITLE-ABS-KEY ( carcinomas, AND endometrial ) OR TITLE-ABS-KEY ( endometrial AND carcinomas ) OR TITLE-ABS-KEY ( endometrial AND cancer ) OR TITLE-ABS-KEY ( cancer, AND endometrial ) OR TITLE-ABS-KEY ( cancers, AND endometrial ) OR TITLE-ABS-KEY ( endometrial AND cancers ) OR TITLE-ABS-KEY ( endometrium AND cancer ) OR TITLE-ABS-KEY ( cancer, AND endometrium ) OR TITLE-ABS-KEY ( cancers, AND endometrium ) OR TITLE-ABS-KEY ( cancer AND of AND the AND endometrium ) OR TITLE-ABS-KEY ( carcinoma AND of AND endometrium ) OR TITLE-ABS-KEY ( endometrium AND carcinoma ) OR TITLE-ABS-KEY ( endometrium AND carcinomas ) OR TITLE-ABS-KEY ( cancer AND of AND endometrium ) OR TITLE-ABS-KEY ( endometrium AND cancers ) )  75,111  #2 ( TITLE-ABS-KEY ( artificial AND intelligence ) OR TITLE-ABS-KEY ( intelligence, AND artificial ) OR TITLE-ABS-KEY ( computational AND intelligence ) OR TITLE-ABS-KEY ( intelligence, AND computational ) OR TITLE-ABS-KEY ( machine AND intelligence ) OR TITLE-ABS-KEY ( intelligence, AND machine ) OR TITLE-ABS-KEY ( computer AND reasoning ) OR TITLE-ABS-KEY ( ai ) OR TITLE-ABS-KEY ( reasoning, AND computer ) OR TITLE-ABS-KEY ( computer AND vision AND systems ) OR TITLE-ABS-KEY ( computer AND vision AND system ) OR TITLE-ABS-KEY ( system, AND computer AND vision ) OR TITLE-ABS-KEY ( systems, AND computer AND vision ) OR TITLE-ABS-KEY ( vision AND system, AND computer ) OR TITLE-ABS-KEY ( vision AND systems, AND computer ) OR TITLE-ABS-KEY ( knowledge AND acquisition ) OR TITLE-ABS-KEY ( acquisition, AND knowledge ) OR TITLE-ABS-KEY ( knowledge AND representation ) OR TITLE-ABS-KEY ( knowledge AND representations ) OR TITLE-ABS-KEY ( representation, AND knowledge ) )  996,807  #3 #2 AND #1 362 | 1st  January  2024 | 362 |
| Web of  science | #1 Endometrial Neoplasms (Topic) or Endometrial Neoplasm (Topic) or Neoplasm, Endometrial (Topic) or Neoplasms, Endometrial (Topic) or Endometrial Carcinoma (Topic) or Carcinoma, Endometrial (Topic) or Carcinomas, Endometrial (Topic) or Endometrial Carcinomas (Topic) or Endometrial Cancer (Topic) or Cancer, Endometrial (Topic) or Cancers, Endometrial (Topic) or Endometrial Cancers (Topic) or Endometrium Cancer (Topic) or Cancer, Endometrium (Topic) or Cancers, Endometrium (Topic) or Cancer of the Endometrium (Topic) or Carcinoma of Endometrium (Topic) or Endometrium Carcinoma (Topic) or Endometrium Carcinomas (Topic) or Cancer of Endometrium (Topic) or Endometrium Cancers (Topic)  81, 466  #2 Artificial Intelligence (Topic) or Intelligence, Artificial (Topic) or Computational Intelligence (Topic) or Intelligence, Computational (Topic) or Machine Intelligence (Topic) or Intelligence, Machine (Topic) or Computer Reasoning (Topic) or Reasoning, Computer (Topic) or AI (Artificial Intelligence) (Topic) or Computer Vision Systems (Topic) or Computer Vision System (Topic) or System, Computer Vision (Topic) or Systems, Computer Vision (Topic) or Vision System, Computer (Topic) or Vision Systems, Computer (Topic) or Knowledge Acquisition (Computer) (Topic) or Acquisition, Knowledge (Computer) (Topic) or Knowledge Representation (Computer) (Topic) or Knowledge Representations (Computer) (Topic) or Representation, Knowledge (Computer) (Topic)  1,192,624  #3 #2 AND #1 264 | 1^st^  January  2024 | 264 |
| Cochrane  library | #1 MeSH descriptor: [Endometrial Neoplasms] explode all trees 1214  #2 Endometrial Neoplasm 606  #3 Neoplasm, Endometrial 606  #4 Neoplasms, Endometrial 1692  #5 Endometrial Carcinoma 958  #6 Carcinoma, Endometrial 958  #7 Carcinomas, Endometrial 87  #8 Endometrial Carcinomas 87  #9 Cancer, Endometrial 2997  #10 Endometrial Cancer 2997  #11 Cancers, Endometrial 508  #12 Endometrial Cancers 508  #13 Endometrium Cancer 1703  #14 Cancer, Endometrium 1703  #15 Cancer, Endometrium 1703  #16 Cancer of the Endometrium 1634  #17 Carcinoma of Endometrium 573  #18 Endometrium Carcinoma 573  #19 Endometrium Carcinomas 46  #20 Cancer of Endometrium 1703  #21 Cancer of Endometrium 1703  #22 #1 OR #2 OR #3 OR #4 OR #5 OR #6 OR #7 OR #8 OR #9 OR #10 OR #11 OR #12 OR #13 OR #14 OR #15 OR #16 OR #17 OR #18 OR #19 OR #20 OR #21 3738  #23 MeSH descriptor: [Artificial Intelligence] explode all trees 3251  #24 Intelligence, Artificial 2471  #25 Computational Intelligence 137  #26 Intelligence, Computational 137  #27 Machine Intelligence 478  #28 Intelligence, Machine 478  #39 Computer Reasoning 385  #30 Reasoning, Computer 385  #31 AI (Artificial Intelligence) 1075  #32 Computer Vision Systems 325  #33 Computer Vision System 763  #34 System, Computer Vision 763  #35 Systems, Computer Vision 325  #36 Vision System, Computer 763  #37 Vision Systems, Computer 325  #38 Knowledge Acquisition (Computer) 464  #39 Acquisition, Knowledge (Computer) 464  #40 Knowledge Representation (Computer) 272  #41 Knowledge Representations (Computer) 61  #42 Representation, Knowledge (Computer) 272  #43 #23 OR #24 OR #25 OR #26 OR #27 OR #28 OR #29 OR #30 OR #31 OR #32 OR #33 OR #34 OR #35 OR #36 OR #37 OR #38 OR #39 OR #40 OR #41 OR #42 6822  #44 #22 AND #43  39 | 1st  January  2024 | 39 |
| Embase | #1 'endometrial neoplasms'/exp OR 'endometrial neoplasms' OR (endometrial AND ('neoplasms'/exp OR neoplasms)) OR (endometrial AND neoplasm) OR (neoplasm, AND endometrial) OR (neoplasms, AND endometrial) OR (endometrial AND carcinoma) OR (carcinoma, AND endometrial) OR (carcinomas, AND endometrial) OR (endometrial AND carcinomas) OR (endometrial AND cancer) OR (cancer, AND endometrial) OR (cancers, AND endometrial) OR (endometrial AND cancers) OR (endometrium AND cancer) OR (cancer, AND endometrium) OR (cancers, AND endometrium) OR (cancer AND of AND the AND endometrium) OR (carcinoma AND of AND endometrium) OR (endometrium AND carcinoma) OR (endometrium AND carcinomas) OR (cancer AND of AND endometrium) OR (endometrium AND cancers) 110,973  #2 'artificial intelligence'/exp OR 'artificial intelligence' OR (artificial AND ('intelligence'/exp OR intelligence)) OR (intelligence, AND artificial) OR (computational AND intelligence) OR (intelligence, AND computational) OR (machine AND intelligence) OR (intelligence, AND machine) OR (computer AND reasoning) OR (reasoning, AND computer) OR (ai AND artificial AND intelligence) OR (computer AND vision AND systems) OR (computer AND vision AND system) OR (system, AND computer AND vision) OR (systems, AND computer AND vision) OR (vision AND system, AND computer) OR (vision AND systems, AND computer) OR (knowledge AND acquisition AND computer) OR (acquisition, AND knowledge AND computer) OR (knowledge AND representation AND computer) OR (knowledge AND representations AND computer) OR (representation, AND knowledge AND computer)  159,699  #3 #2 AND #1 250 | 1st  January  2024 | 250 |

**Table S3. Supplementary Characteristics of Included Studies**

| Author(year) | PPV | NPV | Function and Objectives of the AI System | Source of Training Data | Data-Study Population Match |
| --- | --- | --- | --- | --- | --- |
| Chen et al (2020) | 0.44 | 0.95 | Assesses the depth of MI in EC using deep learning methods. | Single center (Gynecological and Obstetric Hospital, School of Medicine, Fudan University, P. R. China) , 530 cases, MRI data. | Mostly aligned, but lacks diversity. |
| Chiappa et al (2021) | 0.80 | 0.86 | Distinguishes uterine leiomyomas (benign) from uterine sarcomas (malignant). | Single center (Fondazione IRCCS Istituto Nazionale dei Tumori of Milan),70 cases, ultrasound image. | Mostly aligned, but the sample size is small and lacks diversity. |
| Dongli Zhao et al (2022) | 0.98 | 0.23 | Identifies characteristic genes of EC by analyzing differentially expressed genes in public gene expression databases. | Public database (TCGA, GEO), 842 cases, RNA-seq data. | Well-aligned, uses normalized data. |
| Ebrahimian et al (2020) | 0.79 | 0.78 | Classifies pathological images and generates the probability distribution of the main categories. | Public patch-level histopathology dataset, 3,302 cases, image patch. | Mostly aligned, but lacks class balance |
| Fengjun Zhao et al (2022) | 0.99 | 0.89 | Diagnoses various endometrial lesions to improve the efficiency and accuracy of detecting precancerous endometrial conditions. | Single center (Medical Ethics Committee of Northwestern Women’s and Children’s Hospital, China), 602 cases, pathological image. | Mostly aligned, but lacks diversity. |
| Hart et al (2020) | 0.96 | 0.94 | Identifies high-risk women for endometrial cancer based on non-invasive personal health data. | Public dataset (PLCO dataset), 78,215 cases, multivariable health data. | Well-aligned. The dataset includes various potentially relevant health factors and has a large sample size. |
| Li et al (2021) | 0.83 | 0.74 | Distinguishes endometrial cancer from non-cancerous lesions. | Multi-center (three academic institutions), 926 cases, computed tomography imaging data. | Mostly aligned, but lacks of population representativeness |
| Li et al (2022) | 0.20 | 1.00 | Detecting and classifying endometrial cell clusters to distinguish between benign and malignant lesions. | Single center(The First Affiliated Hospital of Xi'an Jiaotong University, China) ,113 cases, pathological image. | Well-aligned. |
| Makris et al (2017) | 0.90 | 0.92 | Classifies endometrial cell nuclei and cases to distinguish between benign and malignant lesions. | Multi-center (Attikon Hospital and St. Savas Cancer Hospital, Greece), 416 cases, cytology samples. | Mostly aligned, but lacks of population representativeness |
| Saida et al (2022) | 0.95 | 0.64 | Differentiates uterine carcinosarcoma from endometrial carcinoma. | Single center(The University of Tsukuba Hospital, Japan), 331 cases, MRI data. | Mostly aligned, but lacks diversity. |
| Sun et al (2020) | 1.00 | 0.92 | Differentiates normal endometrium, endometrial polyps, endometrial hyperplasia, and endometrial adenocarcinoma. | Single center(The Third Affiliated Hospital of Zhengzhou University, China) ,113 cases, pathological image. | Mostly aligned, but lacks diversity. |
| Takahashi et al (2021) | 0.69 | 0.98 | Analyzes an endometrial lesion in a hysteroscopy image. | Single center(The University of Tokyo Hospital,Japan) ,177 cases, hysteroscopy video images. | Mostly aligned. |
| Urushibara et al (2022) | 0.89 | 0.93 | Classifies cases into malignant (endometrial cancer) and non-malignant (including benign lesions and other non-cancerous conditions). | Single center(The University of Tsukuba Hospital, Japan) ,485 cases, MRI data. | Mostly aligned. |
| Abbreviations: MI=myometrial invasion; EC=endometrial cancer; PLCO = Prostate, Lung, Colorectal, and Ovarian Cancer Screening Trial. | | | | | |

**Table S4 – Quality Assessment of Diagnostic Accuracy Studies-2 Tool**

| **DOMAIN 1: Patient Selection**   1. **Risk of Bias**  \| Describe methods of patient selection: \| \| --- \|  - Was a consecutive or random sample of patients enrolled?   Yes/No/Unclear  Yes/No/Unclear  Yes/No/Unclear   - Was a case–control design avoided? - Did the study avoid inappropriate exclusions?   **Could the selection of patients have introduced bias? RISK: LOW/HIGH/UNCLEAR**   1. **Concerns regarding applicability**  \| Describe included patients (previous testing, presentation, intended use of index test, and setting): \| \| --- \|   **Are there concerns that the included patients do not match CONCERN: LOW/HIGH/UNCLEAR**  **the review question?** |
| --- | --- | --- |

| **DOMAIN 2: Index Test**   1. **Risk of Bias**  \| Describe the index test and how it was conducted and interpreted: \| \| --- \|  - Were the index test results interpreted without   Yes/No/Unclear    Yes/No/Unclear  knowledge of the results of the reference standard?   - If a threshold was used, was it prespecified?   **Could the conduct or interpretation of the index test RISK: LOW/HIGH/UNCLEAR**  **have introduced bias?**   1. **Concerns regarding applicability**   **Are there concerns that the index test, its conduct, or CONCERN: LOW/HIGH/UNCLEAR**  **its interpretation differ from the review question?** |
| --- | --- |

| **DOMAIN 3: Reference Standard**   1. **Risk of Bias**  \| Describe the reference standard and how it was conducted and interpreted: \| \| --- \|  - Is the reference standard likely to correctly classify   Yes/No/Unclear    Yes/No/Unclear  the target condition?   - Were the reference standard results interpreted without   knowledge of the results of the index test?  **Could the reference standard, its conduct, or its RISK: LOW/HIGH/UNCLEAR**  **interpretation have introduced bias?**   1. **Concerns regarding applicability**     **Are there concerns that the target condition as defined by CONCERN: LOW/HIGH/UNCLEAR**  **the reference standard does not match the review question?** |
| --- | --- |

| **DOMAIN 4: Flow and Timing**   1. **Risk of Bias**  \| Describe any patients who did not receive the index tests or reference standard or who were excluded from the 2 x 2 table (refer to flow diagram):    Describe the interval and any interventions between index tests and the reference standard: \| \| --- \|  - Was there an appropriate interval between index tests   Yes/No/Unclear  Yes/No/Unclear  Yes/No/Unclear  and reference standard?   - Did all patients receive the same reference standard? - Were all patients included in the analysis?   **Could the patient flow have introduced bias?**  **RISK: LOW/HIGH/UNCLEAR** |
| --- | --- |

**Revised QUADAS-2 Domains with AI-Specific Signaling Questions:**

| Domain | Standard QUADAS-2 Question | AI-Tailored Question | Modification Type | Rationale |
| --- | --- | --- | --- | --- |
| Patient Selection | Case-control design avoided? | Training/test sets strictly separated? | Replace | Avoid bias caused by data breaches |
| Index Test | Threshold pre-specified? | Model code publicly available? | Add | evaluating model reproducibility |

**Table S5. GRADE summary of evidence.**

|  | | Factors that may decrease quality of evidence | | | | |  | | Number per 1,000 tested for given prevalence of target condition | | |  |
| --- | --- | --- | --- | --- | --- | --- | --- | --- | --- | --- | --- | --- |
| Test result | Study design | Risk of bias | Indirectness | Inconsistency | Imprecision | Publication bias | Test Property (95% CI) | Test result | 0.2% | 50% | 100% | Certainty of evidence |
| Sensitivity  (TP +FN) | 13 cohort | Very serious | Very serious | Very Serious | Not serious | Undetected | 0.86 (0.79, 0.90) | TPs | 2 (1, 2) | 430 (395, 450) | 860 (790, 900) | ⨁◯◯◯  Very low |
|  |  |  |  |  |  |  |  | FNs | 0(0, 1) | 70 (50, 105) | 140 (100, 210) |  |
| Specificity (FP + TN) | 13 cohort | Very serious | Very serious3 | Serious | Not serious | Undetected | 0.92 (0.87, 0.95) | FPs | 80 (50, 130) | 40 (25, 65) | 0 | ⨁◯◯◯  Very low |
|  |  |  |  |  |  |  |  | TNs | 918 (868, 948) | 460 (435, 475) | 0 |  |

Abbreviations: CI=Confidence interval; TP=True positive; FN=False negative; FP=False positive; TN=True negative

**Table S6. List of excluded studies**

| **No** | **Study** | **Reason for exclusion** |
| --- | --- | --- |
| 1 | Akazawa, M., & Hashimoto, K. (2021). Artificial intelligence in gynecologic cancers: Current status and future challenges—A systematic review. Artificial Intelligence in Medicine, 120((Akazawa M., navirez@yahoo.co.jp; Hashimoto K.) Department of Obstetrics and Gynecology, Tokyo Women’s Medical University Medical Center East, Tokyo, Japan), 102164. Scopus. https://doi.org/10.1016/j.artmed.2021.102164 | Review |
| 2 | Alam, M. R., Abdul-Ghafar, J., Yim, K., Thakur, N., Lee, S. H., Jang, H.-J., Jung, C. K., & Chong, Y. (2022). Recent Applications of Artificial Intelligence from Histopathologic Image-Based Prediction of Microsatellite Instability in Solid Cancers: A Systematic Review. CANCERS, 14(11). Scopus. https://doi.org/10.3390/cancers14112590 | Review |
| 3 | Bhardwaj, V., Sharma, A., Parambath, S. V., Gul, I., Zhang, X., Lobie, P. E., Qin, P., & Pandey, V. (2022). Machine Learning for Endometrial Cancer Prediction and Prognostication. FRONTIERS IN ONCOLOGY, 12((Bhardwaj V.; Sharma A.; Lobie P.E.; Qin P.; Pandey V., vijay.pandey@sz.tsinghua.edu.cn) Tsinghua Berkeley Shenzhen Institute, Tsinghua Shenzhen International Graduate School, Tsinghua University, Shenzhen, China). Scopus. https://doi.org/10.3389/fonc.2022.852746 | Review |
| 4 | Bhinder, B., Gilvary, C., Madhukar, N. S., & Elemento, O. (2021). Artifi Cial intelligence in cancer research and precision medicine. Cancer Discovery, 11(4), 900–915. Scopus. https://doi.org/10.1158/2159-8290.CD-21-0090 | Review |
| 5 | Davenport, C., Rai, N., Sharma, P., Deeks, J., Berhane, S., Mallett, S., Saha, P., Champaneria, R., Bayliss, S., Snell, K., & et al. (2022). Menopausal status, ultrasound and biomarker tests in combination for the diagnosis of ovarian cancer in symptomatic women. Cochrane Database of Systematic Reviews, 7. https://doi.org/10.1002/14651858.CD011964.pub2 | Review |
| 6 | DeNardis, S. A., Holloway, R. W., Bigsby, G. E. 4th, Pikaart, D. P., Ahmad, S., & Finkler, N. J. (2008). Robotically assisted laparoscopic hysterectomy versus total abdominal hysterectomy and lymphadenectomy for endometrial cancer. Gynecologic Oncology, 111(3), 412–417. https://doi.org/10.1016/j.ygyno.2008.08.025 | Review |
| 7 | Fiste, O., Liontos, M., Zagouri, F., Stamatakos, G., & Dimopoulos, M. A. (2022). Machine learning applications in gynecological cancer: A critical review. Critical Reviews in Oncology/Hematology, 179((Fiste O., ofiste@med.uoa.gr; Liontos M.; Zagouri F.; Dimopoulos M.A.) Department of Clinical Therapeutics, School of Medicine, National and Kapodistrian University of Athens, Alexandra Hospital, 80 Vasilissis Sophias, Athens, Greece). Embase. https://doi.org/10.1016/j.critrevonc.2022.103808 | Review |
| 8 | Guo, Z.-H., Chen, Z.-H., You, Z.-H., Wang, Y.-B., Yi, H.-C., & Wang, M.-N. (2022). A learning-based method to predict LncRNA-disease associations by combining CNN and ELM. BMC Bioinformatics, 22(Suppl 5), 622. https://doi.org/10.1186/s12859-022-04611-3 | Review |
| 9 | Hoivik, E. A., Hodneland, E., Dybvik, J. A., Wagner-Larsen, K. S., Fasmer, K. E., Berg, H. F., Halle, M. K., Haldorsen, I. S., & Krakstad, C. (2021). A radiogenomics application for prognostic profiling of endometrial cancer. Communications Biology, 4(1), 1363. https://doi.org/10.1038/s42003-021-02894-5 | Review |
| 10 | Holloway, R. W., Bravo, R. A. M., Rakowski, J. A., James, J. A., Jeppson, C. N., Ingersoll, S. B., & Ahmad, S. (2012). Detection of sentinel lymph nodes in patients with endometrial cancer undergoing robotic-assisted staging: A comparison of colorimetric and fluorescence imaging. Gynecologic Oncology, 126(1), 25–29. https://doi.org/10.1016/j.ygyno.2012.04.009 | Review |
| 11 | How, J., Lau, S., Press, J., Ferenczy, A., Pelmus, M., Stern, J., Probst, S., Brin, S., Drummond, N., & Gotlieb, W. (2012). Accuracy of sentinel lymph node detection following intra-operative cervical injection for endometrial cancer: A prospective study. Gynecologic Oncology, 127(2), 332–337. https://doi.org/10.1016/j.ygyno.2012.08.018 | Review |
| 12 | Kristensen, S. E., Mosgaard, B. J., Rosendahl, M., Dalsgaard, T., Bjørn, S. F., Frøding, L. P., Kehlet, H., Høgdall, C. K., & Lajer, H. (2017). Robot-assisted surgery in gynecological oncology: Current status and controversies on patient benefits, cost and surgeon conditions—A systematic  review. Acta Obstetricia et Gynecologica Scandinavica, 96(3), 274–285. Scopus. https://doi.org/10.1111/aogs.13084 | Review |
| 13 | Krizova, A., Clarke, B. A., Bernardini, M. Q., James, S., Kalloger, S. E., Boerner, S. L., & Mulligan, A. M. (2011). Histologic artifacts in abdominal, vaginal, laparoscopic, and robotic hysterectomy specimens: A blinded, retrospective review. The American Journal of Surgical Pathology, 35(1), 115–126. https://doi.org/10.1097/PAS.0b013e31820273dc | Review |
| 14 | Lambaudie, E., Houvenaeghel, G., Walz, J., Bannier, M., Buttarelli, M., Gurriet, B., De Laparrent, T., & Blache, J. L. (2008). Robot-assisted laparoscopy in gynecologic oncology. Surgical Endoscopy, 22(12), 2743–2747. https://doi.org/10.1007/s00464-008-0116-5 | Review |
| 15 | Lau, S., Aubin, S., Rosberger, Z., Gourdji, I., How, J., Gotlieb, R., Drummond, N., Eniu, I., Abitbol, J., & Gotlieb, W. (2014). Health-related quality of life following robotic surgery: A pilot study. Journal of Obstetrics and Gynaecology Canada : JOGC = Journal d’obstetrique et Gynecologie Du Canada : JOGC, 36(12), 1071–1078. https://doi.org/10.1016/S1701-2163(15)30384-4 | Review |
| 16 | Lavoue, V., Zeng, X., Lau, S., Press, J. Z., Abitbol, J., Gotlieb, R., How, J., Wang, Y., & Gotlieb, W. H. (2014). Impact of robotics on the outcome of elderly patients with endometrial cancer. Gynecologic Oncology, 133(3), 556–562. https://doi.org/10.1016/j.ygyno.2014.03.572 | Review |
| 17 | Lecointre, L., Dana, J., Lodi, M., Akladios, C., & Gallix, B. (2021). Artificial intelligence-based radiomics models in endometrial cancer: A systematic review. European Journal of Surgical Oncology : The Journal of the European Society of Surgical Oncology and the British Association of Surgical Oncology, 47(11), 2734–2741. Scopus. https://doi.org/10.1016/j.ejso.2021.06.023 | Review |
| 18 | Leitao, M. M. J., Bartashnik, A., Wagner, I., Lee, S. J., Caroline, A., Hoskins, W. J., Thaler, H. T., Abu-Rustum, N. R., Sonoda, Y., Brown, C. L., Jewell, E. L., Barakat, R. R., & Gardner, G. J. (2014). Cost-effectiveness analysis of robotically assisted laparoscopy for newly diagnosed uterine cancers. Obstetrics and Gynecology, 123(5), 1031–1037. https://doi.org/10.1097/AOG.0000000000000223 | Review |
| 19 | Leitao, M. M. J., Malhotra, V., Briscoe, G., Suidan, R., Dholakiya, P., Santos, K., Jewell, E. L., Brown, C. L., Sonoda, Y., Abu-Rustum, N. R., Barakat, R. R., & Gardner, G. J. (2013). Postoperative pain medication requirements in patients undergoing computer-assisted (“Robotic”) and standard laparoscopic procedures for newly  diagnosed endometrial cancer. Annals of Surgical Oncology, 20(11), 3561–3567. https://doi.org/10.1245/s10434-013-3064-9 | Review |
| 20 | Leitao, M. M., Jr., Briscoe, G., Santos, K., Winder, A., Jewell, E. L., Hoskins, W. J., Chi, D. S., Abu-Rustum, N. R., Sonoda, Y., Brown, C. L., Levine, D. A., Barakat, R. R., & Gardner, G. J. (2012). Introduction of a computer-based surgical platform in the surgical care of patients with newly diagnosed uterine cancer: Outcomes and impact on approach. GYNECOLOGIC ONCOLOGY, 125(2), 394–399. https://doi.org/10.1016/j.ygyno.2012.01.046 | Review |
| 21 | Li, W., Qin, Y., Chen, X., & Wang, X. (2023). Mining of clinical and prognosis related genes in the tumor microenvironment of endometrial cancer: A field synopsis of observational study. Medicine, 102(25), e34047. https://doi.org/10.1097/MD.0000000000034047 | Review |
| 22 | Lim, P. C., Kang, E., & Park, D. H. (2011). A comparative detail analysis of the learning curve and surgical outcome for robotic hysterectomy with lymphadenectomy versus laparoscopic hysterectomy with  lymphadenectomy in treatment of endometrial cancer: A case-matched controlled  study of the first one hundred twenty two patients. Gynecologic Oncology, 120(3), 413–418. https://doi.org/10.1016/j.ygyno.2010.11.034 | Review |
| 23 | Liu, S., Zeng, C., Lv, H., Zhang, Y., Xiong, H., & Tang, H. (2022). A Novel Defined Pyroptosis-Related Gene Signature for Predicting the Prognosis of Endometrial Cancer. Disease Markers, 2022, 7570494. https://doi.org/10.1155/2022/7570494 | Review |
| 24 | Liu, X., Gao, J., Wang, J., You, J., Chu, J., & Jin, Z. (2022). Esthetics Effect and the Modified Placement of Robotic-Assisted Single-Site Laparoscopic Gynecologic Surgery by Common Robotic Instruments and LAGIS  Single-Site Port. Journal of Investigative Surgery : The Official Journal of the Academy of Surgical Research, 35(2), 434–439. https://doi.org/10.1080/08941939.2020.1845880 | Review |
| 25 | Lönnerfors, C., Bossmar, T., & Persson, J. (2013). Port-site metastases following robot-assisted laparoscopic surgery for gynecological malignancies. Acta Obstetricia et Gynecologica Scandinavica, 92(12), 1361–1368. https://doi.org/10.1111/aogs.12245 | Review |
| 26 | López-Reig, R., Fernández-Serra, A., Romero, I., Zorrero, C., Illueca, C., García-Casado, Z., Poveda, A., & López-Guerrero, J. A. (2019). Prognostic classification of endometrial cancer using a molecular approach based on a twelve-gene NGS panel. Scientific Reports, 9(1), 18093. Medline. https://doi.org/10.1038/s41598-019-54624-x | Review |
| 27 | Mateva, S., Nikolova, M., & Yordanov, A. (2021). Patterns of Myometrial Invasion in Endometrial Adenocarcinoma with Emphasizing on Microcystic, Elongated and Fragmented (MELF) Glands Pattern: A Narrative Review of the Literature. DIAGNOSTICS, 11(9). https://doi.org/10.3390/diagnostics11091707 | Review |
| 28 | Mekala, V. R., Hui-Shan, C., Jan-Gowth, C., & Ng, K.-L. (2022). Identification of Key Prognosis-related microRNAs in Early-and Late-Stage Gynecological Cancers Based on TCGA Data. Current Bioinformatics, 17(9), 860–872. Embase. https://doi.org/10.2174/1574893617666220802154148 | Review |
| 29 | Mello, A. C., Freitas, M., Coutinho, L., Falcon, T., & Matte, U. (2020). Machine Learning Supports Long Noncoding RNAs as Expression Markers for Endometrial Carcinoma. BioMed Research International, 2020, 3968279. https://doi.org/10.1155/2020/3968279 | Review |
| 30 | Morland, D., Triumbari, E. K. A., Boldrini, L., Gatta, R., Pizzuto, D., & Annunziata, S. (2022). Radiomics in Oncological PET Imaging: A Systematic Review—Part 2, Infradiaphragmatic Cancers, Blood Malignancies, Melanoma and Musculoskeletal Cancers. Diagnostics, 12(6). Scopus. https://doi.org/10.3390/diagnostics12061330 | Review |
| 31 | Mysona, D. P., Kapp, D. S., Rohatgi, A., Lee, D., Mann, A. K., Tran, P., Tran, L., She, J.-X., & Chan, J. K. (2021). Applying Artificial Intelligence to Gynecologic Oncology: A Review. Obstetrical and Gynecological Survey, 76(5), 292–301. Scopus. https://doi.org/10.1097/ogx.0000000000000902 | Review |
| 32 | Ouyang, D., Li, R., Li, Y., & Zhu, X. (2019). Construction of a Competitive Endogenous RNA Network in Uterine Corpus Endometrial Carcinoma. Medical Science Monitor : International Medical Journal of Experimental and Clinical Research, 25, 7998–8010. https://doi.org/10.12659/MSM.915798 | Review |
| 33 | Park, J. H., Kim, E. Y., Luchini, C., Eccher, A., Tizaoui, K., Shin, J. I., & Lim, B. J. (2022). Artificial Intelligence for Predicting Microsatellite Instability Based on Tumor Histomorphology: A Systematic Review. International Journal of Molecular Sciences, 23(5). Scopus. https://doi.org/10.3390/ijms23052462 | Review |
| 34 | Paulino, E., & de Melo, A. C. (2020). Adjuvant treatment of endometrial cancer in molecular era: Are we ready to move on? CRITICAL REVIEWS IN ONCOLOGY HEMATOLOGY, 153. https://doi.org/10.1016/j.critrevonc.2020.103016 | Review |
| 35 | Pernikářová, V., & Bouchal, P. (2015). Targeted proteomics of solid cancers: From quantification of known biomarkers towards reading the digital proteome maps. Expert Review of Proteomics, 12(6), 651–667. Scopus. https://doi.org/10.1586/14789450.2015.1094381 | Review |
| 36 | Peungjesada, S., Bhosale, P. R., Balachandran, A., & Iyer, R. B. (2009). Magnetic Resonance Imaging of Endometrial Carcinoma. JOURNAL OF COMPUTER ASSISTED TOMOGRAPHY, 33(4), 601–608. https://doi.org/10.1097/RCT.0b013e31818d4279 | Review |
| 37 | Qi, N., Zhang, Z., Xiang, Y., & Harrington, P. de B. (2012). Locally linear embedding method for dimensionality reduction of tissue sections of endometrial carcinoma by near infrared spectroscopy. Analytica Chimica Acta, 724, 12–19. https://doi.org/10.1016/j.aca.2012.02.040 | Review |
| 38 | Ravegnini, G., Ferioli, M., Pantaleo, M. A., Morganti, A. G., De Leo, A., De Iaco, P., Rizzo, S., & Perrone, A. M. (2022). Radiomics and artificial intelligence in malignant uterine body cancers: Protocol for a systematic review. PloS One, 17(6 June), e0267727. Scopus. https://doi.org/10.1371/journal.pone.0267727 | Review |
| 39 | Robova, H., Rob, L., Halaska, M. J., Pluta, M., & Skapa, P. (2013). Current status of sentinel lymph node mapping in the management of endometrial cancer. Expert Review of Anticancer Therapy, 13(1), 55–61. https://doi.org/10.1586/era.12.157 | Review |
| 40 | Roman, H., Quibel, S., Auber, M., Muszynski, H., Huet, E., Marpeau, L., & Tuech, J. J. (2015). Recurrences and fertility after endometrioma ablation in women with and without colorectal endometriosis: A prospective cohort study. HUMAN REPRODUCTION, 30(3), 558–568. https://doi.org/10.1093/humrep/deu354 | Review |
| 41 | Romani, C., Calza, S., Todeschini, P., Tassi, R. A., Zanotti, L., Bandiera, E., Sartori, E., Pecorelli, S., Ravaggi, A., Santin, A. D., & Bignotti, E. (2014). Identification of optimal reference genes for gene expression normalization in a wide cohort of endometrioid endometrial carcinoma tissues. PLoS ONE, 9(12). Embase. https://doi.org/10.1371/journal.pone.0113781 | Review |
| 42 | Seamon, L. G., Cohn, D. E., Richardson, D. L., Valmadre, S., Carlson, M. J., Phillips, G. S., & Fowler, J. M. (2008). Robotic hysterectomy and pelvic-aortic lymphadenectomy for endometrial cancer. Obstetrics and Gynecology, 112(6), 1207–1213. https://doi.org/10.1097/AOG.0b013e31818e4416 | Review |
| 43 | Sharma, A., & Menon, U. (2006). Screening for gynaecological cancers. European Journal of Surgical Oncology, 32(8), 818–824. Scopus. https://doi.org/10.1016/j.ejso.2006.03.034 | Review |
| 44 | Shrestha, P., Poudyal, B., Yadollahi, S., E Wright, D., V Gregory, A., D Warner, J., Korfiatis, P., C Green, I., L Rassier, S., Mariani, A., Kim, B., Laughlin-Tommaso, S. K., & L Kline, T. (2022). A systematic review on the use of artificial intelligence in gynecologic imaging—Background, state of the art, and future directions. Gynecologic Oncology, 166(3), 596–605. Scopus. https://doi.org/10.1016/j.ygyno.2022.07.024 | Review |
| 45 | Sideris, M., Darwish, A., Rallis, K., Emin, E. I., & Mould, T. (2021). Prognostic biomarkers for atypical endometrial hyperplasia: A mini review. International Journal of Gynecological Cancer, 31(SUPPL 1), A337. Embase. https://doi.org/10.1136/ijgc-2021-ESGO.596 | Review |
| 46 | Sobocinska, J., Kolenda, T., Teresiak, A., Badziag-Lesniak, N., Kopczynska, M., Guglas, K., Przybyla, A., Filas, V., Bogajewska-Rylko, E., Lamperska, K., & Mackiewicz, A. (2020). Diagnostics of Mutations in MMR/EPCAM Genes and Their Role in the Treatment and Care of Patients with Lynch Syndrome. DIAGNOSTICS, 10(10). https://doi.org/10.3390/diagnostics10100786 | Review |
| 47 | Sone, K., Toyohara, Y., Taguchi, A., Miyamoto, Y., Tanikawa, M., Uchino-Mori, M., Iriyama, T., Tsuruga, T., & Osuga, Y. (2021). Application of artificial intelligence in gynecologic malignancies: A review. The Journal of Obstetrics and Gynaecology Research, 47(8), 2577–2585. Scopus. https://doi.org/10.1111/jog.14818 | Review |
| 48 | Stahl, J. M., Park, H. S., Silasi, D.-A., Azodi, M., & Damast, S. (2016). Influence of robotic-assisted laparoscopic hysterectomy on vaginal cuff healing and brachytherapy initiation in endometrial carcinoma patients. Practical Radiation Oncology, 6(4), 226–232. https://doi.org/10.1016/j.prro.2015.09.015 | Review |
| 49 | Strasser-Weippl, K., Badovinac-Crnjevic, T., Fan, L., & Goss, P. E. (2013). Extended adjuvant endocrine therapy in hormone-receptor positive breast cancer. Breast, 22(S2), S171–S175. Scopus. https://doi.org/10.1016/j.breast.2013.07.033 | Review |
| 50 | Van den Heuvel, F., Han, I., Chungbin, S., Strowbridge, A., Tekyi-Mensah, S., & Ragan, D. (1999). Development and clinical implementation of an enhanced display algorithm for use in networked electronic portal imaging. INTERNATIONAL JOURNAL OF RADIATION ONCOLOGY BIOLOGY PHYSICS, 45(4), 1041–1053. https://doi.org/10.1016/S0360-3016(99)00291-6 | Review |
| 51 | Vidyasagar, M. (2017). Machine learning methods in computational cancer biology. ANNUAL REVIEWS IN CONTROL, 43, 107–127. Scopus. https://doi.org/10.1016/j.arcontrol.2017.03.007 | Review |
| 52 | Villasco, A., & D’Alonzo, M. (2020). Extended endocrine therapy in premenopausal breast cancer patients: Where are we now? Breast Journal, 26(10), 2018–2020. Scopus. https://doi.org/10.1111/tbj.13895 | Review |
| 53 | Wang, J., Li, S., Yu, L., Qu, A., Wang, Q., Liu, J., & Wu, Q. (2023). SDPN: A Slight Dual-Path Network with Local-Global Attention Guided for Medical Image Segmentation. IEEE Journal of Biomedical and Health Informatics, 27(6), 2956–2967. Embase. https://doi.org/10.1109/JBHI.2023.3260026 | Review |
| 54 | Wang, M., Li, L., Liu, J., & Wang, J. (2018). A gene interaction network‑based method to measure the common and heterogeneous mechanisms of gynecological cancer. Molecular Medicine Reports, 18(1), 230–242. https://doi.org/10.3892/mmr.2018.8961 | Review |
| 55 | Wang, S., & Huo, X. (2021). Comprehensive Analysis of ESRRA in Endometrial Cancer. Technology in Cancer Research & Treatment, 20, 1533033821992083. https://doi.org/10.1177/1533033821992083 | Review |
| 56 | Wu, J. Q., Horeweg, N., de Bruyn, M., Nout, R. A., Jürgenliemk-Schulz, I. M., Lutgens, L. C. H. W., Jobsen, J. J., van der Steen-Banasik, E. M., Nijman, H. W., Smit, V. T. H. B. M., Bosse, T., Creutzberg, C. L., & Koelzer, V. H. (2022). Automated causal inference in application to randomized controlled clinical trials. Nature Machine Intelligence, 4(5), 436–444. Scopus. https://doi.org/10.1038/s42256-022-00470-y | Review |
| 57 | Wu, M., Yan, C., Liu, H., & Liu, Q. (2018). Automatic classification of ovarian cancer types from cytological images using deep convolutional neural networks. Bioscience Reports, 38(3), BSR20180289. https://doi.org/10.1042/BSR20180289 | Review |
| 58 | Wu, Q., Zhang, N., & Xie, X. (2022). The clinicopathological characteristics of POLE-mutated/ultramutated endometrial carcinoma and prognostic value of POLE status: A meta-analysis based on 49 articles incorporating 12,120 patients. BMC Cancer, 22(1). Scopus. https://doi.org/10.1186/s12885-022-10267-2 | Review |
| 59 | Xiao Wen, Yang Lu, Pan Feng, Cao Run-yu, Yao Tian, & Li Xiao-ping. (2018). Automatic Phase Aberration Compensation for Digital Holographic Microscopy Combined with Phase Fitting and Deep Learning. ACTA PHOTONICA SINICA, 47(12). https://doi.org/10.3788/gzxb20184712.1210001 | Review |
| 60 | Yang, S., Bi, J., Drnevich, J., Li, K., & Nowak, R. A. (2022). Basigin is necessary for normal decidualization of human uterine stromal cells. HUMAN REPRODUCTION, 37(12), 2885–2898. https://doi.org/10.1093/humrep/deac229 | Review |
| 61 | Yao, Y., Chen, Y., Wang, Y., Li, X., Wang, J., Shen, D., & Wei, L. (2010). Molecular classification of human endometrial cancer based on gene expression profiles from specialized microarrays. International Journal of Gynecology and Obstetrics, 110(2), 125–129. Embase. https://doi.org/10.1016/j.ijgo.2010.03.020 | Review |
| 62 | Zhao, M., Liu, Y., & O’Mara, T. A. (2016). ECGene: A Literature-Based Knowledgebase of Endometrial Cancer Genes. Human Mutation, 37(4), 337–343. https://doi.org/10.1002/humu.22950 | Review |
| 63 | Zhao, X., Xia, X., Wang, X., Bai, M., Zhan, D., & Shu, K. (2022). Deep Learning-Based Protein Features Predict Overall Survival and Chemotherapy Benefit in Gastric Cancer. Frontiers in Oncology, 12((Zhao X.; Bai M.; Shu K., shukx@cqupt.edu.cn) Chongqing Key Laboratory of Big Data for Bio Intelligence, School of Bioinformation, Chongqing University of Posts and Telecommunications, Chongqing, China). Embase. https://doi.org/10.3389/fonc.2022.847706 | Review |
| 64 | Zhu, X. H., Li, X. M., Zhang, W. L., Liao, M. M., Li, Y., Wang, F. F., Shang, B., Peng, L. G., Su, Y. J., You, Z. J., Shi, J. Y., Zhong, W. L., Liang, X. R., Liang, C. J., Liang, L., Liao, W. T., & Ding, Y. Q. (2021). [Application of artificial intelligence-assisted diagnosis for cervical liquid-based thin-layer cytology]. Zhonghua bing li xue za zhi = Chinese journal of pathology, 50(4), 333–338. Medline. https://doi.org/10.3760/cma.j.cn112151-20201013-00780 | Review |
| 65 | Zhu, X., Ying, J., Yang, H., Fu, L., Li, B., & Jiang, B. (2021). Detection of deep myometrial invasion in endometrial cancer MR imaging based on multi-feature fusion and probabilistic support vector machine ensemble. Computers in Biology and Medicine, 134, 104487. https://doi.org/10.1016/j.compbiomed.2021.104487 | Review |
| 66 | Akazawa, M., Hashimoto, K., Noda, K., & Yoshida, K. (2021). The application of machine learning for predicting recurrence in patients with early-stage endometrial cancer: a pilot study. Obstetrics & gynecology science, 64(3), 266–273. https://doi.org/10.5468/ogs.20248 | Without relevant outcomes |
| 67 | AlShibli, A., & Mathkour, H. (2019). A Shallow Convolutional Learning Network for Classification of Cancers Based on Copy Number Variations. Sensors (Basel, Switzerland), 19(19), 4207. https://doi.org/10.3390/s19194207 | Without relevant outcomes |
| 68 | Asami, Y., Hiranuma, K., Takayanagi, D., Matsuda, M., Shimada, Y., Kato, M. K., Kuno, I., Murakami, N., Komatsu, M., Hamamoto, R., Kohno, T., Sekizawa, A., Matsumoto, K., Kato, T., Yoshida, H., & Shiraishi, K. (2022). Predictive model for the preoperative assessment and prognostic modeling of lymph node metastasis in endometrial cancer. Scientific reports, 12(1), 19004. https://doi.org/10.1038/s41598-022-23252-3 | Without relevant outcomes |
| 69 | Aşıcıoğlu, O., Gungorduk, K., Ozdemir, A., Güngördük, Ö., Gokçü, M., Yaşar, L., & Sancı, M. (2019). A novel preoperative scoring system based on 18-FDG PET-CT for predicting lymph node metastases in patients with high-risk endometrial cancer. Journal of obstetrics and gynaecology : the journal of the Institute of Obstetrics and Gynaecology, 39(1), 105–109. https://doi.org/10.1080/01443615.2018.1467884 | Without relevant outcomes |
| 70 | Chang, C. Y., Lu, Y. A., Ting, W. C., Shen, T. D., & Peng, W. C. (2021). An artificial immune system with bootstrap sampling for the diagnosis of recurrent endometrial cancers. Open medicine (Warsaw, Poland), 16(1), 237–245. https://doi.org/10.1515/med-2021-0226 | Without relevant outcomes |
| 71 | David, S., Plante, A., Dallaire, F., Tremblay, J. P., Sheehy, G., Macdonald, E., Forrest, L., Daneshmand, M., Trudel, D., Wilson, B. C., Hopkins, L., Murugkar, S., Vanderhyden, B., & Leblond, F. (2022). Multispectral label-free Raman spectroscopy can detect ovarian and endometrial cancer with high accuracy. Journal of biophotonics, 15(2), e202100198. https://doi.org/10.1002/jbio.202100198 | Without relevant outcomes |
| 72 | Dong, H. C., Dong, H. K., Yu, M. H., Lin, Y. H., & Chang, C. C. (2020). Using Deep Learning with Convolutional Neural Network Approach to Identify the Invasion Depth of Endometrial Cancer in Myometrium Using MR Images: A Pilot Study. International journal of environmental research and public health, 17(16), 5993. https://doi.org/10.3390/ijerph17165993 | Without relevant outcomes |
| 73 | Erdemoglu, E., Serel, T. A., Karacan, E., Köksal, O. K., Turan, İ., Öztürk, V., & Bozkurt, K. K. (2023). Artificial intelligence for prediction of endometrial intraepithelial neoplasia and endometrial cancer risks in pre- and postmenopausal women. AJOG global reports, 3(1), 100154. https://doi.org/10.1016/j.xagr.2022.100154 | Without relevant outcomes |
| 74 | Fell, C., Mohammadi, M., Morrison, D., Arandjelović, O., Syed, S., Konanahalli, P., Bell, S., Bryson, G., Harrison, D. J., & Harris-Birtill, D. (2023). Detection of malignancy in whole slide images of endometrial cancer biopsies using artificial intelligence. PloS one, 18(3), e0282577. https://doi.org/10.1371/journal.pone.0282577 | Without relevant outcomes |
| 75 | Feng, M., Zhao, Y., Chen, J., Zhao, T., Mei, J., Fan, Y., Lin, Z., Yao, J., & Bu, H. (2023). A deep learning model for lymph node metastasis prediction based on digital histopathological images of primary endometrial cancer. *Quantitative imaging in medicine and surgery*, *13*(3), 1899–1913. https://doi.org/10.21037/qims-22-220 | Without relevant outcomes |
| 76 | Günakan, E., Atan, S., Haberal, A. N., Küçükyıldız, İ. A., Gökçe, E., & Ayhan, A. (2019). A novel prediction method for lymph node involvement in endometrial cancer: machine learning. *International journal of gynecological cancer : official journal of the International Gynecological Cancer Society*, *29*(2), 320–324. https://doi.org/10.1136/ijgc-2018-000033 | Without relevant outcomes |
| 77 | Houri, O., Gil, Y., Gemer, O., Helpman, L., Vaknin, Z., Lavie, O., Arie, A. B., Amit, A., Levy, T., Namazov, A., Shachar, I. B., Atlas, I., Bruchim, I., & Eitan, R. (2022). Prediction of endometrial cancer recurrence by using a novel machine learning algorithm: An Israeli gynecologic oncology group study. *Journal of gynecology obstetrics and human reproduction*, *51*(9), 102466. https://doi.org/10.1016/j.jogoh.2022.102466 | Without relevant outcomes |
| 78 | Hu, H., Wang, H., Bai, Y., & Liu, M. (2019). Determination of endometrial carcinoma with gene expression based on optimized Elman neural network. *Appl. Math. Comput., 341*, 204-214. | Without relevant outcomes |
| 79 | Ladbury, C., Li, R., Shiao, J., Liu, J., Cristea, M., Han, E., Dellinger, T., Lee, S., Wang, E., Fisher, C., Chen, Y. J., Amini, A., Robin, T., & Glaser, S. (2022). Characterizing impact of positive lymph node number in endometrial cancer using machine-learning: A better prognostic indicator than FIGO staging?. *Gynecologic oncology*, *164*(1), 39–45. https://doi.org/10.1016/j.ygyno.2021.11.007 | Without relevant outcomes |
| 80 | Liao, X., Li, Q., Zheng, X., & He, J. (2021). Computer-Aided Decision-Making System for Endometrial Atypical Hyperplasia based on Multimodal & Multi-Instance Deep Convolution Networks. | Without relevant outcomes |
| 81 | Lin, G., Ng, K. K., Chang, C. J., Wang, J. J., Ho, K. C., Yen, T. C., Wu, T. I., Wang, C. C., Chen, Y. R., Huang, Y. T., Ng, S. H., Jung, S. M., Chang, T. C., & Lai, C. H. (2009). Myometrial invasion in endometrial cancer: diagnostic accuracy of diffusion-weighted 3.0-T MR imaging--initial experience. *Radiology*, *250*(3), 784–792. https://doi.org/10.1148/radiol.2503080874 | Without relevant outcomes |
| 82 | Lin, Y., Song, H., Zheng, R., Cai, J., Li, Z., & Li, H. (2019). Computer-aided classification system for early endometrial cancer of co-registered photoacoustic and ultrasonic signals (Vol. 11190). SPIE. https://doi.org/10.1117/12.2536709 | Without relevant outcomes |
| 83 | Liu, X., Jin, S., & Zi, D. (2023). Overall survival prediction models for gynecological endometrioid adenocarcinoma with squamous differentiation (GE-ASqD) using machine-learning algorithms. *Scientific reports*, *13*(1), 8395. https://doi.org/10.1038/s41598-023-33748-1 | Without relevant outcomes |
| 84 | Mainenti, P. P., Stanzione, A., Cuocolo, R., Del Grosso, R., Danzi, R., Romeo, V., Raffone, A., Di Spiezio Sardo, A., Giordano, E., Travaglino, A., Insabato, L., Scaglione, M., Maurea, S., & Brunetti, A. (2022). MRI radiomics: A machine learning approach for the risk stratification of endometrial cancer patients. *European journal of radiology*, *149*, 110226. https://doi.org/10.1016/j.ejrad.2022.110226 | Without relevant outcomes |
| 85 | W. Mao, L. Xiong, Z. Li and Y. Lin, "Transfer Learning-Based Detection of Endometrial Cancer Lesion Regions on MRI Images," 2022 IEEE 2nd International Conference on Software Engineering and Artificial Intelligence (SEAI), Xiamen, China, 2022, pp. 46-49, doi: 10.1109/SEAI55746.2022.9832165. | Without relevant outcomes |
| 86 | Marcus, D., Phelps, D. L., Savage, A., Balog, J., Kudo, H., Dina, R., Bodai, Z., Rosini, F., Ip, J., Amgheib, A., Abda, J., Manoli, E., McKenzie, J., Yazbek, J., Takats, Z., & Ghaem-Maghami, S. (2022). Point-of-Care Diagnosis of Endometrial Cancer Using the Surgical Intelligent Knife (iKnife)-A Prospective Pilot Study of Diagnostic Accuracy. *Cancers*, *14*(23), 5892. https://doi.org/10.3390/cancers14235892 | Without relevant outcomes |
| 87 | Moro, F., Albanese, M., Boldrini, L., Chiappa, V., Lenkowicz, J., Bertolina, F., Mascilini, F., Moroni, R., Gambacorta, M. A., Raspagliesi, F., Scambia, G., Testa, A. C., & Fanfani, F. (2022). Developing and validating ultrasound-based radiomics models for predicting high-risk endometrial cancer. *Ultrasound in obstetrics & gynecology : the official journal of the International Society of Ultrasound in Obstetrics and Gynecology*, *60*(2), 256–268. https://doi.org/10.1002/uog.24805 | Without relevant outcomes |
| 88 | Otani, S., Himoto, Y., Nishio, M., Fujimoto, K., Moribata, Y., Yakami, M., Kurata, Y., Hamanishi, J., Ueda, A., Minamiguchi, S., Mandai, M., & Kido, A. (2022). Radiomic machine learning for pretreatment assessment of prognostic risk factors for endometrial cancer and its effects on radiologists' decisions of deep myometrial invasion. *Magnetic resonance imaging*, *85*, 161–167. https://doi.org/10.1016/j.mri.2021.10.024 | Without relevant outcomes |
| 89 | Pandey, M., & Gromiha, M. M. (2023). MutBLESS: A tool to identify disease-prone sites in cancer using deep learning. *Biochimica et biophysica acta. Molecular basis of disease*, *1869*(6), 166721. https://doi.org/10.1016/j.bbadis.2023.166721 | Without relevant outcomes |
| 90 | Parlatan, U., Inanc, M. T., Ozgor, B. Y., Oral, E., Bastu, E., Unlu, M. B., & Basar, G. (2019). Raman spectroscopy as a non-invasive diagnostic technique for endometriosis. *Scientific reports*, *9*(1), 19795. https://doi.org/10.1038/s41598-019-56308-y | Without relevant outcomes |
| 91 | Perez-Sanchez, C., Colas, E., Cabrera, S., Falcon, O., Sanchez-del-Río, A., García, E., Fernández-de-Castillo, L., Muruzabal, J. C., Alvarez, E., Fiol, G., González, C., Torrejón, R., Moral, E., Campos, M., Repollés, M., Carreras, R., Jiménez-López, J., Xercavins, J., Aibar, E., Perdones-Montero, A., … Reventós, J. (2013). Molecular diagnosis of endometrial cancer from uterine aspirates. *International journal of cancer*, *133*(10), 2383–2391. https://doi.org/10.1002/ijc.28243 | Without relevant outcomes |
| 92 | Pergialiotis, V., Pouliakis, A., Parthenis, C., Damaskou, V., Chrelias, C., Papantoniou, N., & Panayiotides, I. (2018). The utility of artificial neural networks and classification and regression trees for the prediction of endometrial cancer in postmenopausal women. *Public health*, *164*, 1–6. https://doi.org/10.1016/j.puhe.2018.07.012 | Without relevant outcomes |
| 93 | Piedimonte, S., Feigenberg, T., Drysdale, E., Kwon, J., Gotlieb, W. H., Cormier, B., Plante, M., Lau, S., Helpman, L., Renaud, M. C., May, T., & Vicus, D. (2022). Predicting recurrence and recurrence-free survival in high-grade endometrial cancer using machine learning. *Journal of surgical oncology*, *126*(6), 1096–1103. https://doi.org/10.1002/jso.27008 | Without relevant outcomes |
| 94 | Pouliakis, A., Margari, C., Margari, N., Chrelias, C., Zygouris, D., Meristoudis, C., Panayiotides, I., & Karakitsos, P. (2014). Using classification and regression trees, liquid-based cytology and nuclear morphometry for the discrimination of endometrial lesions. *Diagnostic cytopathology*, *42*(7), 582–591. https://doi.org/10.1002/dc.23077 | Without relevant outcomes |
| 95 | Praiss, A. M., Huang, Y., St Clair, C. M., Tergas, A. I., Melamed, A., Khoury-Collado, F., Hou, J. Y., Hu, J., Hur, C., Hershman, D. L., & Wright, J. D. (2020). Using machine learning to create prognostic systems for endometrial cancer. *Gynecologic oncology*, *159*(3), 744–750. https://doi.org/10.1016/j.ygyno.2020.09.047 | Without relevant outcomes |
| 96 | Reijnen, C., Gogou, E., Visser, N. C. M., Engerud, H., Ramjith, J., van der Putten, L. J. M., van de Vijver, K., Santacana, M., Bronsert, P., Bulten, J., Hirschfeld, M., Colas, E., Gil-Moreno, A., Reques, A., Mancebo, G., Krakstad, C., Trovik, J., Haldorsen, I. S., Huvila, J., Koskas, M., … Pijnenborg, J. M. A. (2020). Preoperative risk stratification in endometrial cancer (ENDORISK) by a Bayesian network model: A development and validation study. *PLoS medicine*, *17*(5), e1003111. https://doi.org/10.1371/journal.pmed.1003111 | Without relevant outcomes |
| 97 | Rewcastle, E., Gudlaugsson, E., Lillesand, M., Skaland, I., Baak, J. P. A., & Janssen, E. A. M. (2023). Automated Prognostic Assessment of Endometrial Hyperplasia for Progression Risk Evaluation Using Artificial Intelligence. *Modern pathology : an official journal of the United States and Canadian Academy of Pathology, Inc*, *36*(5), 100116. https://doi.org/10.1016/j.modpat.2023.100116 | Without relevant outcomes |
| 98 | Rodríguez-Ortega, A., Alegre, A., Lago, V., Carot-Sierra, J. M., Ten-Esteve, A., Montoliu, G., Domingo, S., Alberich-Bayarri, Á., & Martí-Bonmatí, L. (2021). Machine Learning-Based Integration of Prognostic Magnetic Resonance Imaging Biomarkers for Myometrial Invasion Stratification in Endometrial Cancer. Journal of magnetic resonance imaging : JMRI, 54(3), 987–995. https://doi.org/10.1002/jmri.27625 | Without relevant outcomes |
| 99 | Shazly, S. A., Coronado, P. J., Yılmaz, E., Melekoglu, R., Sahin, H., Giannella, L., Ciavattini, A., Carpini, G. D., Di Giuseppe, J., Yordanov, A., Karakadieva, K., Nedelcheva, N. M., Vasileva-Slaveva, M., Alcazar, J. L., Chacon, E., Manzour, N., Vara, J., Karaman, E., Karaaslan, O., Hacıoğlu, L., … Middle-Eastern College of Obstetricians and Gynaecologists (MCOG) Muti-Center Studies (MCS) office and Artificial Intelligence Unit (AI) (2023). Endometrial Cancer Individualized Scoring System (ECISS): A machine learning-based prediction model of endometrial cancer prognosis. *International journal of gynaecology and obstetrics: the official organ of the International Federation of Gynaecology and Obstetrics*, *161*(3), 760–768. https://doi.org/10.1002/ijgo.14639 | Without relevant outcomes |
| 100 | Song, J., Im, S., Lee, S. H., & Jang, H. J. (2022). Deep Learning-Based Classification of Uterine Cervical and Endometrial Cancer Subtypes from Whole-Slide Histopathology Images. Diagnostics (Basel, Switzerland), 12(11), 2623. https://doi.org/10.3390/diagnostics12112623 | Without relevant outcomes |
| 101 | Tao, J., Wang, Y., Liang, Y., & Zhang, A. (2022). Evaluation and Monitoring of Endometrial Cancer Based on Magnetic Resonance Imaging Features of Deep Learning. *Contrast media & molecular imaging*, *2022*, 5198592. https://doi.org/10.1155/2022/5198592 | Without relevant outcomes |
| 102 | Veeraraghavan, H., Friedman, C. F., DeLair, D. F., Ninčević, J., Himoto, Y., Bruni, S. G., Cappello, G., Petkovska, I., Nougaret, S., Nikolovski, I., Zehir, A., Abu-Rustum, N. R., Aghajanian, C., Zamarin, D., Cadoo, K. A., Diaz, L. A., Jr, Leitao, M. M., Jr, Makker, V., Soslow, R. A., Mueller, J. J., … Lakhman, Y. (2020). Machine learning-based prediction of microsatellite instability and high tumor mutation burden from contrast-enhanced computed tomography in endometrial cancers. *Scientific reports*, *10*(1), 17769. https://doi.org/10.1038/s41598-020-72475-9 | Without relevant outcomes |
| 103 | Wang, W., Xu, Y., Yuan, S., Li, Z., Zhu, X., Zhou, Q., Shen, W., & Wang, S. (2022). Prediction of Endometrial Carcinoma Using the Combination of Electronic Health Records and an Ensemble Machine Learning Method. *Frontiers in medicine*, *9*, 851890. https://doi.org/10.3389/fmed.2022.851890 | Without relevant outcomes |
| 104 | Wu, E., Fan, X., Tang, T., Li, J., Wang, J., Liu, X., Zungar, Z., Ren, J., Wu, C., & Shen, B. (2022). Biomarkers discovery for endometrial cancer: A graph convolutional sample network method. *Computers in biology and medicine*, *150*, 106200. https://doi.org/10.1016/j.compbiomed.2022.106200 | Without relevant outcomes |
| 105 | Xia, Z., Zhang, L., Liu, S. *et al.* Deep learning-based hysteroscopic intelligent examination and ultrasound examination for diagnosis of endometrial carcinoma. *J Supercomput* **78**, 11229–11244 (2022). https://doi.org/10.1007/s11227-021-04046-2 | Without relevant outcomes |
| 106 | Xu, X., Li, H., Wang, S., Fang, M., Zhong, L., Fan, W., Dong, D., Tian, J., & Zhao, X. (2019). Multiplanar MRI-Based Predictive Model for Preoperative Assessment of Lymph Node Metastasis in Endometrial Cancer. *Frontiers in oncology*, *9*, 1007. https://doi.org/10.3389/fonc.2019.01007 | Without relevant outcomes |
| 107 | Yang, F., Tian, J., Xiang, Y., Zhang, Z., & Harrington, P.deB. (2012). Near infrared spectroscopy combined with least squares support vector machines and fuzzy rule-building expert system applied to diagnosis of endometrial carcinoma. *Cancer epidemiology*, *36*(3), 317–323. https://doi.org/10.1016/j.canep.2011.10.009 | Without relevant outcomes |
| 108 | Yin, F., Shao, X., Zhao, L., Li, X., Zhou, J., Cheng, Y., He, X., Lei, S., Li, J., & Wang, J. (2019). Predicting prognosis of endometrioid endometrial adenocarcinoma on the basis of gene expression and clinical features using Random Forest. *Oncology letters*, *18*(2), 1597–1606. https://doi.org/10.3892/ol.2019.10504 | Without relevant outcomes |
| 109 | Zhang, H., Qureshi, M. A., Wahid, M., Charifa, A., Ehsan, A., Ip, A., De Dios, I., Ma, W., Sharma, I., McCloskey, J., Donato, M., Siegel, D., Gutierrez, M., Pecora, A., Goy, A., & Albitar, M. (2023). Differential Diagnosis of Hematologic and Solid Tumors Using Targeted Transcriptome and Artificial Intelligence. *The American journal of pathology*, *193*(1), 51–59. https://doi.org/10.1016/j.ajpath.2022.09.006 | Without relevant outcomes |
| 110 | Zhang, X., Ba, W., Zhao, X., Wang, C., Li, Q., Zhang, Y., Lu, S., Wang, L., Wang, S., Song, Z., & Shen, D. (2022). Clinical-grade endometrial cancer detection system *via* whole-slide images using deep learning. *Frontiers in oncology*, *12*, 1040238. https://doi.org/10.3389/fonc.2022.1040238 | Without relevant outcomes |
| 111 | Zhang, Y., Gong, C., Zheng, L., Li, X., & Yang, X. (2021). Deep Learning for Intelligent Recognition and Prediction of Endometrial Cancer. *Journal of healthcare engineering*, *2021*, 1148309. https://doi.org/10.1155/2021/1148309 | Without relevant outcomes |
| 112 | Zhang, Y., Wang, Z., Zhang, J., Wang, C., Wang, Y., Chen, H., Shan, L., Huo, J., Gu, J., & Ma, X. (2021). Deep learning model for classifying endometrial lesions. *Journal of translational medicine*, *19*(1), 10. https://doi.org/10.1186/s12967-020-02660-x | Without relevant outcomes |
| 113 | Zhu, X., Ying, J., Yang, H., Fu, L., Li, B., & Jiang, B. (2021). Detection of deep myometrial invasion in endometrial cancer MR imaging based on multi-feature fusion and probabilistic support vector machine ensemble. *Computers in biology and medicine*, *134*, 104487. https://doi.org/10.1016/j.compbiomed.2021.104487 | Without relevant outcomes |
| 114 | Lombana Merlano, R., Moreno, A., & Dorado, J. (1975). Epidemiologic aspects in gynecology obstetrics. Screening for carcinoma of the uterine cervix in 1,109 women. Revista Colombiana de Obstetricia y Ginecología, 26(2), 125 - 132. | Not computer-aided detection |
| 115 | Krill, L. S., & Bristow, R. E. (2013). Robotic surgery: gynecologic oncology. Cancer Journal (Sudbury, Mass.), 19(2), 167 - 176. https://doi.org/10.1097/PPO.0b013e31828a3293 | Not computer-aided detection |
| 116 | Baak J. P. (1995). The role of computerized morphometric and cytometric feature analysis in endometrial hyperplasia and cancer prognosis. *Journal of cellular biochemistry. Supplement*, *23*, 137–146. https://doi.org/10.1002/jcb.240590918 | Data cannot be extracted |
| 117 | Barczyński, B., Frąszczak, K., Wnorowski, A., & Kotarski, J. (2023). Menopausal Status Contributes to Overall Survival in Endometrial Cancer Patients. *Cancers*, *15*(2), 451. https://doi.org/10.3390/cancers15020451 | Data cannot be extracted |
| 118 | Bowditch, R. C., Clarke, J. M., Baird, P. J., & Greenberg, M. L. (2015). Morphologic analysis of false negative SurePath® slides using Focalpoint™ GS computer-assisted cervical screening technology: An Australian experience. *Diagnostic cytopathology*, *43*(11), 870–878. https://doi.org/10.1002/dc.23314 | Data cannot be extracted |
| 119 | Chiappa, V., Interlenghi, M., Bogani, G., Salvatore, C., Bertolina, F., Sarpietro, G., Signorelli, M., Ronzulli, D., Castiglioni, I., & Raspagliesi, F. (2021). A decision support system based on radiomics and machine learning to predict the risk of malignancy of ovarian masses from transvaginal ultrasonography and serum CA-125. *European radiology experimental*, *5*(1), 28. https://doi.org/10.1186/s41747-021-00226-0 | Data cannot be extracted |
| 120 | Delanerolle, G., Yang, X., Shetty, S., Raymont, V., Shetty, A., Phiri, P., Hapangama, D. K., Tempest, N., Majumder, K., & Shi, J. Q. (2021). Artificial intelligence: A rapid case for advancement in the personalization of Gynaecology/Obstetric and Mental Health care. *Women's health (London, England)*, *17*, 17455065211018111. https://doi.org/10.1177/17455065211018111 | Data cannot be extracted |
| 121 | DeStephano, C. C., Bakkum-Gamez, J. N., Kaunitz, A. M., Ridgeway, J. L., & Sherman, M. E. (2020). Intercepting Endometrial Cancer: Opportunities to Expand Access Using New Technology. *Cancer prevention research (Philadelphia, Pa.)*, *13*(7), 563–568. https://doi.org/10.1158/1940-6207.CAPR-19-0556 | Data cannot be extracted |
| 122 | Díaz-Gimeno, P., Ruiz-Alonso, M., Blesa, D., Bosch, N., Martínez-Conejero, J. A., Alamá, P., Garrido, N., Pellicer, A., & Simón, C. (2013). The accuracy and reproducibility of the endometrial receptivity array is superior to histology as a diagnostic method for endometrial receptivity. *Fertility and sterility*, *99*(2), 508–517. https://doi.org/10.1016/j.fertnstert.2012.09.046 | Data cannot be extracted |
| 123 | Downing, M. J., Papke, D. J., Jr, Tyekucheva, S., & Mutter, G. L. (2020). A New Classification of Benign, Premalignant, and Malignant Endometrial Tissues Using Machine Learning Applied to 1413 Candidate Variables. *International journal of gynecological pathology : official journal of the International Society of Gynecological Pathologists*, *39*(4), 333–343. https://doi.org/10.1097/PGP.0000000000000615 | Data cannot be extracted |
| 124 | Eitan, R., Sabah, G., Krissi, H., Raban, O., Ben-Haroush, A., Goldschmit, C., Levavi, H., & Peled, Y. (2015). Robotic blue-dye sentinel lymph node detection for endometrial cancer - Factors predicting successful mapping. *European journal of surgical oncology : the journal of the European Society of Surgical Oncology and the British Association of Surgical Oncology*, *41*(12), 1659–1663. https://doi.org/10.1016/j.ejso.2015.09.006 | Data cannot be extracted |
| 125 | Farres, A., Teixeira, N., Espanol, P., Magret, E., Luna, R., Soler, C., Martin-Malpartida, P., Macías, M. J., Céspedes, M. V., Rovira, R. (2022). 2022 - LBA - 1382 - ESGO Genomic signatures for the prediction of recurrence and metastasis in Endometrial Cancer. International Journal of Gynecological Cancer, 32(Supplement 2), A471. ISSN 1048 - 891X. https://doi.org/10.1136/ijgc - 2022 - ESGO.1016 (https://www.sciencedirect.com/science/article/pii/S1048891X24128435) | Data cannot be extracted |
| 126 | Fechete, R., Morar, I. A., Moldovan, D., Chelcea, R. I., Crainic, R., & Nicoară, S. C. (2021). Fourier and Laplace-like low-field NMR spectroscopy: The perspectives of multivariate and artificial neural networks analyses. *Journal of magnetic resonance (San Diego, Calif. : 1997)*, *324*, 106915. https://doi.org/10.1016/j.jmr.2021.106915 | Data cannot be extracted |
| 127 | Feng, Y., Wang, Z., Xiao, M., Li, J., Su, Y., Delvoux, B., Zhang, Z., Dekker, A., Xanthoulea, S., Zhang, Z., Traverso, A., Romano, A., Zhang, Z., Liu, C., Gao, H., Wang, S., & Qian, L. (2022). An Applicable Machine Learning Model Based on Preoperative Examinations Predicts Histology, Stage, and Grade for Endometrial Cancer. *Frontiers in oncology*, *12*, 904597. https://doi.org/10.3389/fonc.2022.904597 | Data cannot be extracted |
| 128 | Finan, M. A., Harris, J. A., Fisher, A. M., Bradley, K., Henslee, H., & Rocconi, R. P. (2012). Magnetic resonance or computerized tomography imaging to predict difficulty of robotic surgery for endometrial cancer. *Journal of robotic surgery*, *6*(2), 131–137. https://doi.org/10.1007/s11701-011-0281-8 | Data cannot be extracted |
| 129 | Fremond, S., Andani, S., Barkey Wolf, J., Dijkstra, J., Melsbach, S., Jobsen, J. J., Brinkhuis, M., Roothaan, S., Jurgenliemk-Schulz, I., Lutgens, L. C. H. W., Nout, R. A., van der Steen-Banasik, E. M., de Boer, S. M., Powell, M. E., Singh, N., Mileshkin, L. R., Mackay, H. J., Leary, A., Nijman, H. W., Smit, V. T. H. B. M., … Bosse, T. (2023). Interpretable deep learning model to predict the molecular classification of endometrial cancer from haematoxylin and eosin-stained whole-slide images: a combined analysis of the PORTEC randomised trials and clinical cohorts. *The Lancet. Digital health*, *5*(2), e71–e82. https://doi.org/10.1016/S2589-7500(22)00210-2 | Data cannot be extracted |
| 130 | Fremond, S., Koelzer, V. H., Horeweg, N., & Bosse, T. (2022). The evolving role of morphology in endometrial cancer diagnostics: From histopathology and molecular testing towards integrative data analysis by deep learning. *Frontiers in oncology*, *12*, 928977. https://doi.org/10.3389/fonc.2022.928977 | Data cannot be extracted |
| 131 | Geitung J. T. (2021). Editorial for "Machine Learning-Based Integration of Prognostic MR Imaging Biomarkers for Myometrial Invasion Stratification in Endometrial Cancer". *Journal of magnetic resonance imaging : JMRI*, *54*(3), 996. https://doi.org/10.1002/jmri.27750 | Data cannot be extracted |
| 132 | Gomez, P. (2022). Mutational Analysis and Deep Learning Classification of Uterine and Cervical Cancers. bioRxiv. https://doi.org/10.1101/2022.10.12.511895 | Data cannot be extracted |
| 133 | Gupta, P., Gupta, N., & Dey, P. (2016). Artificial Neural Network for Cytodiagnosis of Endometrial Carcinoma. Analytical and Quantitative Cytopathology and Histopathology, 38(4), 249 - 254. ISSN 0884 - 6812 | Data cannot be extracted |
| 134 | Hajjo, R., Sabbah, D. A., Bardaweel, S. K., & Tropsha, A. (2021). Identification of Tumor-Specific MRI Biomarkers Using Machine Learning (ML). *Diagnostics (Basel, Switzerland)*, *11*(5), 742. https://doi.org/10.3390/diagnostics11050742 | Data cannot be extracted |
| 135 | He, L., Wang, Y., Yang, Y., Huang, L., & Wen, Z. (2014). Identifying the gene signatures from gene-pathway bipartite network guarantees the robust model performance on predicting the cancer prognosis. *BioMed research international*, *2014*, 424509. https://doi.org/10.1155/2014/424509 | Data cannot be extracted |
| 136 | Hodneland, E., Dybvik, J. A., Wagner-Larsen, K. S., Šoltészová, V., Munthe-Kaas, A. Z., Fasmer, K. E., Krakstad, C., Lundervold, A., Lundervold, A. S., Salvesen, Ø., Erickson, B. J., & Haldorsen, I. (2021). Automated segmentation of endometrial cancer on MR images using deep learning. *Scientific reports*, *11*(1), 179. https://doi.org/10.1038/s41598-020-80068-9 | Data cannot be extracted |
| 137 | Hong, R., Liu, W., DeLair, D., Razavian, N., & Fenyö, D. (2021). Predicting endometrial cancer subtypes and molecular features from histopathology images using multi-resolution deep learning models. *Cell reports. Medicine*, *2*(9), 100400. https://doi.org/10.1016/j.xcrm.2021.100400 | Data cannot be extracted |
| 138 | Horeweg, N., de Bruyn, M., Nout, R. A., Stelloo, E., Kedziersza, K., León-Castillo, A., Plat, A., Mertz, K. D., Osse, M., Jürgenliemk-Schulz, I. M., Lutgens, L. C. H. W., Jobsen, J. J., van der Steen-Banasik, E. M., Smit, V. T., Creutzberg, C. L., Bosse, T., Nijman, H. W., Koelzer, V. H., & Church, D. N. (2020). Prognostic Integrated Image-Based Immune and Molecular Profiling in Early-Stage Endometrial Cancer. *Cancer immunology research*, *8*(12), 1508–1519. https://doi.org/10.1158/2326-6066.CIR-20-0149 | Data cannot be extracted |
| 139 | Iwai, K., Shigetomi, H., Oka, K., & Kobayashi, H. (2019). Office diagnostic smart hysterofiberscopy, hysmartscopy, using mobile technology: A single center experience and analysis of diagnostic accuracy. World Academy of Sciences Journal, 1(5), 247 - 253. https://doi.org/10.3892/wasj.2019.26 | Data cannot be extracted |
| 140 | Kalra, S., Tizhoosh, H. R., Shah, S., Choi, C., Damaskinos, S., Safarpoor, A., Shafiei, S., Babaie, M., Diamandis, P., Campbell, C. J. V., & Pantanowitz, L. (2020). Pan-cancer diagnostic consensus through searching archival histopathology images using artificial intelligence. *NPJ digital medicine*, *3*, 31. https://doi.org/10.1038/s41746-020-0238-2 | Data cannot be extracted |
| 141 | Kawai, E., Benoit, L., Hotton, J., Rance, B., Bonsang-Kitzis, H., Lécuru, F., Balaya, V., & Ngô, C. (2021). Impact of obesity on surgical and oncologic outcomes in patients with endometrial cancer treated with a robotic approach. *The journal of obstetrics and gynaecology research*, *47*(1), 128–136. https://doi.org/10.1111/jog.14442 | Data cannot be extracted |
| 142 | Kayser, K., & Höffgen, H. (1984). Pattern recognition in histopathology by orders of textures. *Medical informatics = Medecine et informatique*, *9*(1), 55–59. https://doi.org/10.3109/14639238409010938 | Data cannot be extracted |
| 143 | Kehl, K. L., Xu, W., Gusev, A., Bakouny, Z., Choueiri, T. K., Riaz, I. B., Elmarakeby, H., Van Allen, E. M., & Schrag, D. (2021). Artificial intelligence-aided clinical annotation of a large multi-cancer genomic dataset. *Nature communications*, *12*(1), 7304. https://doi.org/10.1038/s41467-021-27358-6 | Data cannot be extracted |
| 144 | Kim, S. I., Chung, J. Y., Paik, H., Seol, A., Yoon, S. H., Kim, T. M., Kim, H. S., Chung, H. H., Cho, J. Y., Kim, J. W., & Lee, M. (2021). Prognostic role of computed tomography-based, artificial intelligence-driven waist skeletal muscle volume in uterine endometrial carcinoma. *Insights into imaging*, *12*(1), 192. https://doi.org/10.1186/s13244-021-01134-y | Data cannot be extracted |
| 145 | Kiranmai, T. S., & Lakshmi, P. V. (2022). A novel whale optimized TGV-FCMS segmentation with modified LSTM classification for endometrium cancer prediction. Indian Journal of Computer Science and Engineering, 13(3), 812 - 826. https://doi.org/10.21817/indjcse/2022/v13i3/221303006 | Data cannot be extracted |
| 146 | Kleppe, A., Albregtsen, F., Vlatkovic, L., Pradhan, M., Nielsen, B., Hveem, T. S., Askautrud, H. A., Kristensen, G. B., Nesbakken, A., Trovik, J., Wæhre, H., Tomlinson, I., Shepherd, N. A., Novelli, M., Kerr, D. J., & Danielsen, H. E. (2018). Chromatin organisation and cancer prognosis: a pan-cancer study. *The Lancet. Oncology*, *19*(3), 356–369. https://doi.org/10.1016/S1470-2045(17)30899-9 | Data cannot be extracted |
| 147 | Kobayashi, M., Kobayashi, H., Nakayama, S., & Adachi, H. (2021). Robot-assisted laparoscopic hysterectomy for endometrial cancer in a patient with Herlyn-Werner-Wunderlich syndrome. *BMJ case reports*, *14*(5), e240001. https://doi.org/10.1136/bcr-2020-240001 | Data cannot be extracted |
| 148 | Kurata, Y., Nishio, M., Kido, A., Fujimoto, K., Yakami, M., Isoda, H., & Togashi, K. (2019). Automatic segmentation of the uterus on MRI using a convolutional neural network. *Computers in biology and medicine*, *114*, 103438. https://doi.org/10.1016/j.compbiomed.2019.103438 | Data cannot be extracted |
| 149 | Kurata, Y., Nishio, M., Moribata, Y., Kido, A., Himoto, Y., Otani, S., Fujimoto, K., Yakami, M., Minamiguchi, S., Mandai, M., & Nakamoto, Y. (2021). Automatic segmentation of uterine endometrial cancer on multi-sequence MRI using a convolutional neural network. *Scientific reports*, *11*(1), 14440. https://doi.org/10.1038/s41598-021-93792-7 | Data cannot be extracted |
| 150 | Lal, A., Panos, R., Marjanovic, M., Walker, M., Fuentes, E., Kapp, D. S., Henner, W. D., Buturovic, L. J., & Halks-Miller, M. (2012). A gene expression profile test for the differential diagnosis of ovarian versus endometrial cancers. *Oncotarget*, *3*(2), 212–223. https://doi.org/10.18632/oncotarget.450 | Data cannot be extracted |
| 151 | Lefebvre, T. L., Ciga, O., Bhatnagar, S. R., Ueno, Y., Saif, S., Winter-Reinhold, E., Dohan, A., Soyer, P., Forghani, R., Siddiqi, K., Seuntjens, J., Reinhold, C., & Savadjiev, P. (2023). Predicting histopathology markers of endometrial carcinoma with a quantitative image analysis approach based on spherical harmonics in multiparametric MRI. *Diagnostic and interventional imaging*, *104*(3), 142–152. https://doi.org/10.1016/j.diii.2022.10.007 | Data cannot be extracted |
| 152 | Lim, P. C., Kang, E., & Park, D. H. (2010). Learning curve and surgical outcome for robotic-assisted hysterectomy with lymphadenectomy: case-matched controlled comparison with laparoscopy and laparotomy for treatment of endometrial cancer. *Journal of minimally invasive gynecology*, *17*(6), 739–748. https://doi.org/10.1016/j.jmig.2010.07.008 | Data cannot be extracted |
| 153 | Lin, G. (2021). Artificial intelligence in gynaecology oncology imaging. Cancer Imaging, 21(SUPPL 1). https://doi.org/10.1186/s40644-021-00422-6 | Data cannot be extracted |
| 154 | Loeffler, C. M. L., El Nahhas, O. S. M., Muti, H. S., Seibel, T., Cifci, D., van Treeck, M., Gustav, M., Carrero, Z. I., Gaisa, N. T., Lehmann, K. V., Leary, A., Selenica, P., Reis-Filho, J. S., Bruechle, N. O., & Kather, J. N. (2023). Direct prediction of Homologous Recombination Deficiency from routine histology in ten different tumor types with attention-based Multiple Instance Learning: a development and validation study. *medRxiv : the preprint server for health sciences*, 2023.03.08.23286975. https://doi.org/10.1101/2023.03.08.23286975 | Data cannot be extracted |
| 155 | Mohammadi, M., Cooper, J., Arandelović, O., Fell, C., Morrison, D., Syed, S., Konanahalli, P., Bell, S., Bryson, G., Harrison, D. J., & Harris-Birtill, D. (2022). Weakly supervised learning and interpretability for endometrial whole slide image diagnosis. *Experimental biology and medicine (Maywood, N.J.)*, *247*(22), 2025–2037. https://doi.org/10.1177/15353702221126560 | Data cannot be extracted |
| 156 | Mushlin, R. A., Gallagher, S., Kershenbaum, A., & Rebbeck, T. R. (2009). Clique-finding for heterogeneity and multidimensionality in biomarker epidemiology research: the CHAMBER algorithm. *PloS one*, *4*(3), e4862. https://doi.org/10.1371/journal.pone.0004862 | Data cannot be extracted |
| 157 | Neofytou, M. S., Tanos, V., Constantinou, I., Kyriacou, E. C., Pattichis, M. S., & Pattichis, C. S. (2015). Computer-aided diagnosis in hysteroscopic imaging. *IEEE journal of biomedical and health informatics*, *19*(3), 1129–1136. https://doi.org/10.1109/JBHI.2014.2332760 | Data cannot be extracted |
| 158 | Neofytou, M. S., Tanos, V., Pattichis, M. S., Pattichis, C. S., Kyriacou, E. C., & Pavlopoulos, S. (2007). Color based texture--classification of hysteroscopy images of the endometrium. *Annual International Conference of the IEEE Engineering in Medicine and Biology Society. IEEE Engineering in Medicine and Biology Society. Annual International Conference*, *2007*, 864–867. https://doi.org/10.1109/IEMBS.2007.4352427 | Data cannot be extracted |
| 159 | Njoku, K., Sutton, C. J., Whetton, A. D., & Crosbie, E. J. (2020). Metabolomic Biomarkers for Detection, Prognosis and Identifying Recurrence in Endometrial Cancer. *Metabolites*, *10*(8), 314. https://doi.org/10.3390/metabo10080314 | Data cannot be extracted |
| 160 | Norimatsu, Y., Irino, S., Maeda, Y., Yanoh, K., Kurokawa, T., Hirai, Y., Kobayashi, T. K., & Fulciniti, F. (2021). Nuclear morphometry as an adjunct to cytopathologic examination of endometrial brushings on LBC samples: A prospective approach to combined evaluation in endometrial neoplasms and look alikes. *Cytopathology : official journal of the British Society for Clinical Cytology*, *32*(1), 65–74. https://doi.org/10.1111/cyt.12902 | Data cannot be extracted |
| 161 | Onisko, A., Druzdzel, M. J., & Austin, R. M. (2019). Application of Bayesian network modeling to pathology informatics. *Diagnostic cytopathology*, *47*(1), 41–47. https://doi.org/10.1002/dc.23993 | Data cannot be extracted |
| 162 | Parlatan, U., Inanc, M. T., Ozgor, B. Y., Oral, E., Bastu, E., Unlu, M. B., & Basar, G. (2019). Raman spectroscopy as a non-invasive diagnostic technique for endometriosis. *Scientific reports*, *9*(1), 19795. https://doi.org/10.1038/s41598-019-56308-y | Data cannot be extracted |
| 163 | Pergialiotis, V., Pouliakis, A., Damaskou, V., Chrelias, C., Kalantaridou, S., & Panayiotides, I. (2019). Incorporating artificial intelligence techniques in decision making concerning the optimal management of postmenopausal women with evidence of endometrial pathology. International Journal of Gynecological Cancer, 29, A105. https://doi.org/10.1136/ijgc-2019-ESGO.145 | Data cannot be extracted |
| 164 | Pinochet, P., Eude, F., Becker, S., Shah, V., Sibille, L., Toledano, M. N., Modzelewski, R., Vera, P., & Decazes, P. (2021). Evaluation of an Automatic Classification Algorithm Using Convolutional Neural Networks in Oncological Positron Emission Tomography. *Frontiers in medicine*, *8*, 628179. https://doi.org/10.3389/fmed.2021.628179 | Data cannot be extracted |
| 165 | Pirone, D., Xin, L., Bianco, V., Miccio, L., & Xiao, W (2023). Identification of drug-resistant cancer cells in flow cytometry combining 3D holographic tomography with machine learning. Sensors and Actuators B: Chemical, 375. https://doi.org/10.1016/j.snb.2022.132963 | Data cannot be extracted |
| 166 | Plotkin, A., Kuzeljevic, B., De Villa, V., Thompson, E. F., Gilks, C. B., Clarke, B. A., Köbel, M., & McAlpine, J. N. (2020). Interlaboratory Concordance of ProMisE Molecular Classification of Endometrial Carcinoma Based on Endometrial Biopsy Specimens. *International journal of gynecological pathology : official journal of the International Society of Gynecological Pathologists*, *39*(6), 537–545. https://doi.org/10.1097/PGP.0000000000000654 | Data cannot be extracted |
| 167 | Pouliakis, A., Margari, C., Margari, N., Chrelias, C., Zygouris, D., Meristoudis, C., Panayiotides, I., & Karakitsos, P. (2014). Using classification and regression trees, liquid-based cytology and nuclear morphometry for the discrimination of endometrial lesions. *Diagnostic cytopathology*, *42*(7), 582–591. https://doi.org/10.1002/dc.23077 | Data cannot be extracted |
| 168 | Pouryahya, M., Oh, J. H., Javanmard, P., Mathews, J. C., Belkhatir, Z., Deasy, J. O., & Tannenbaum, A. R. (2022). aWCluster: A Novel Integrative Network-Based Clustering of Multiomics for Subtype Analysis of Cancer Data. *IEEE/ACM transactions on computational biology and bioinformatics*, *19*(3), 1472–1483. https://doi.org/10.1109/TCBB.2020.3039511 | Data cannot be extracted |
| 169 | Qilin, Z., Peng, B., Ang, Q., Weijuan, J., Ping, J., Hongqing, Z., Bin, D., & Ruijie, Y. (2022). The feasibility study on the generalization of deep learning dose prediction model for volumetric modulated arc therapy of cervical cancer. *Journal of applied clinical medical physics*, *23*(6), e13583. https://doi.org/10.1002/acm2.13583 | Data cannot be extracted |
| 170 | Riasatian, A., Babaie, M., Maleki, D., Kalra, S., Valipour, M., Hemati, S., Zaveri, M., Safarpoor, A., Shafiei, S., Afshari, M., Rasoolijaberi, M., Sikaroudi, M., Adnan, M., Shah, S., Choi, C., Damaskinos, S., Campbell, C. J., Diamandis, P., Pantanowitz, L., Kashani, H., … Tizhoosh, H. R. (2021). Fine-Tuning and training of densenet for histopathology image representation using TCGA diagnostic slides. *Medical image analysis*, *70*, 102032. https://doi.org/10.1016/j.media.2021.102032 | Data cannot be extracted |
| 171 | Rossi, E. C., Ivanova, A., & Boggess, J. F. (2012). Robotically assisted fluorescence-guided lymph node mapping with ICG for gynecologic malignancies: a feasibility study. *Gynecologic oncology*, *124*(1), 78–82. https://doi.org/10.1016/j.ygyno.2011.09.025 | Data cannot be extracted |
| 172 | Rossi, E. C., Jackson, A., Ivanova, A., & Boggess, J. F. (2013). Detection of sentinel nodes for endometrial cancer with robotic assisted fluorescence imaging: cervical versus hysteroscopic injection. *International journal of gynecological cancer : official journal of the International Gynecological Cancer Society*, *23*(9), 1704–1711. https://doi.org/10.1097/IGC.0b013e3182a616f6 | Data cannot be extracted |
| 173 | Abel, J., Jain, S., Rajan, D., Padigela, H., Leidal, K., Prakash, A., Conway, J., Nercessian, M., Kirkup, C., Javed, S. A., Biju, R., Harguindeguy, N., Shenker, D., Indorf, N., Sanghavi, D., Egger, R., Trotter, B., Gerardin, Y., Brosnan-Cashman, J. A., Dhoot, A., … Taylor-Weiner, A. (2024). AI powered quantification of nuclear morphology in cancers enables prediction of genome instability and prognosis. *NPJ precision oncology*, *8*(1), 134. https://doi.org/10.1038/s41698-024-00623-9 | Data cannot be extracted |
| 174 | Shibata, M., Okamura, K., Yura, K., & Umezawa, A. (2020). High-precision multiclass cell classification by supervised machine learning on lectin microarray data. *Regenerative therapy*, *15*, 195–201. https://doi.org/10.1016/j.reth.2020.09.005 | Data cannot be extracted |
| 175 | Shin, S. J., You, S. C., Jeon, H., Jung, J. W., An, M. H., Park, R. W., & Roh, J. (2021). Style transfer strategy for developing a generalizable deep learning application in digital pathology. *Computer methods and programs in biomedicine*, *198*, 105815. https://doi.org/10.1016/j.cmpb.2020.105815 | Data cannot be extracted |
| 176 | Stanzione, A., Cuocolo, R., Del Grosso, R., Nardiello, A., Romeo, V., Travaglino, A., Raffone, A., Bifulco, G., Zullo, F., Insabato, L., Maurea, S., & Mainenti, P. P. (2021). Deep Myometrial Infiltration of Endometrial Cancer on MRI: A Radiomics-Powered Machine Learning Pilot Study. *Academic radiology*, *28*(5), 737–744. https://doi.org/10.1016/j.acra.2020.02.028 | Data cannot be extracted |
| 177 | Sturgis, C. D., Isoe, C., McNeal, N. E., Yu, G. H., & DeFrias, D. V. (1998). PAPNET computer-aided rescreening for detection of benign and malignant glandular elements in cervicovaginal smears: a review of 61 cases. *Diagnostic cytopathology*, *18*(4), 307–311. https://doi.org/10.1002/(sici)1097-0339(199804)18:4<307::aid-dc12>3.0.co;2-n | Data cannot be extracted |
| 178 | Taxt, T., Lundervold, A., Fuglaas, B., Lien, H., & Abeler, V. (1992). Multispectral analysis of uterine corpus tumors in magnetic resonance imaging. *Magnetic resonance in medicine*, *23*(1), 55–76. https://doi.org/10.1002/mrm.1910230108 | Data cannot be extracted |
| 179 | Tian, S., Chen, A., Li, Y., Wang, N., Ma, C., Lin, L., Wang, J., & Liu, A. (2023). The combined application of amide proton transfer imaging and diffusion kurtosis imaging for differentiating stage Ia endometrial carcinoma and endometrial polyps. *Magnetic resonance imaging*, *99*, 67–72. https://doi.org/10.1016/j.mri.2022.12.026 | Data cannot be extracted |
| 180 | Tian, S., Liu, A., Guo, Y., Lin, T., Chen, L., Wang, N., & Li, X. (2022). Tumor global texture analysis based on enhanced T2* weighted angiography sequence R2* map for predicting microsatellite instability of endometrial carcinoma. Chinese Journal of Medical Imaging Technology, 38(2), 257 - 261. https://doi.org/10.13929/j.issn.1003 - 3289.2022.02.022 | Data cannot be extracted |
| 181 | Ugarov, I., Chernyh, V., Sharkova, I., Ivanov, N., Maslennikov, V., Ostapenko, D., & Solovey, V. (2020). The use of artificial intelligence to assess factors of genetic predisposition to cancer of the uterine body. European Journal of Human Genetics, 28(SUPPL 1), 954 - 954. https://doi.org/10.1038/s41431-020-00741-5 | Data cannot be extracted |
| 182 | Vidyasagar M. (2014). Machine learning methods in the computational biology of cancer. *Proceedings. Mathematical, physical, and engineering sciences*, *470*(2167), 20140081. https://doi.org/10.1098/rspa.2014.0081 | Data cannot be extracted |
| 183 | Vigo, F., Tozzi, A., Disler, M., Kavvadias, V., Fedier, A., Heinzelmann-Schwarz, V., & Kavvadias, T.(2022). Gynecological cancer detection using Fourier-transformed infrared spectroscopy in urine samples: Potential and accuracy of machine learning processing. International Journal of Gynecological Cancer, 32, A417. https://doi.org/10.1136/ijgc-2022-ESGO.894 | Data cannot be extracted |
| 184 | Vigo, F., Tozzi, A., Kavvadias, V., Disler, M., & Kavvadias, T.(2022). 6P Urine spectroscopy coupled with artificial intelligence: Proof of concept for a new diagnostic tool to detect gynaecological cancers. Annals of Oncology, 33, S384 - S385. https://doi.org/10.1016/j.annonc.2022.04.024 | Data cannot be extracted |
| 185 | Vlachokosta, A. A., Asvestas, P. A., Gkrozou, F., Lavasidis, L., Matsopoulos, G. K., & Paschopoulos, M. (2013). Classification of hysteroscopical images using texture and vessel descriptors. *Medical & biological engineering & computing*, *51*(8), 859–867. https://doi.org/10.1007/s11517-013-1058-1 | Data cannot be extracted |
| 186 | Vlachokosta, A. A., Asvestas, P. A., Matsopoulos, G. K., Kondi-Pafiti, A., & Vlachos, N. (2013). Classification of histological images of the endometrium using texture features. *Analytical and quantitative cytopathology and histopathology*, *35*(2), 105–113. | Data cannot be extracted |
| 187 | Wang, G., Ma, M., Zhang, Z., Xiang, Y., & Harrington, P.deB. (2013). A novel DPSO-SVM system for variable interval selection of endometrial tissue sections by near infrared spectroscopy. *Talanta*, *112*, 136–142. https://doi.org/10.1016/j.talanta.2013.03.016 | Data cannot be extracted |
| 188 | Wang, H., Xu, Z., Zhang, H., Huang, J., Peng, H., Zhang, Y., Liang, C., Zhao, K., & Liu, Z. (2022). The value of magnetic resonance imaging-based tumor shape features for assessing microsatellite instability status in endometrial cancer. *Quantitative imaging in medicine and surgery*, *12*(9), 4402–4413. https://doi.org/10.21037/qims-22-77 | Data cannot be extracted |
| 189 | Xia, Z., Zhang, L., Liu, S., Ran, W., Liu, Y., & Tu, J. (2020). WITHDRAWN: Analysis of Clinical Stage and Nerve Damage of Endometrial Carcinoma Diagnosed by Hysteroscopy of Artificial Intelligence Computer Information. *Neuroscience letters*, 135215. Advance online publication. https://doi.org/10.1016/j.neulet.2020.135215 | Data cannot be extracted |
| 190 | Xiang, Y., Xu, K., Zhang, Z., Dai, Y., & Harrington, P.deB. (2010). Near-infrared spectroscopic applications for diagnosis of endometrial carcinoma. *Journal of biomedical optics*, *15*(6), 067002. https://doi.org/10.1117/1.3512183 | Data cannot be extracted |
| 191 | Zhai, W., Xiang, Y. H., Dai, Y. M., Zhang, J. J., & Zhang, Z. Y. (2011). *Guang pu xue yu guang pu fen xi = Guang pu*, *31*(4), 932–936. | Data cannot be extracted |
| 192 | Zhang, J. J., Zhang, Z. Y., Xiang, Y. H., & Yang, F. (2013). *Guang pu xue yu guang pu fen xi = Guang pu*, *33*(2), 344–348. | Data cannot be extracted |
| 193 | Zhang Z. Y. (2015). *Guang pu xue yu guang pu fen xi = Guang pu*, *35*(9), 2388–2392. | Data cannot be extracted |
| 194 | Zhao, Y., Pan, Z., Namburi, S., Pattison, A., Posner, A., Balachander, S., Paisie, C. A., Reddi, H. V., Rueter, J., Gill, A. J., Fox, S., Raghav, K. P. S., Flynn, W. F., Tothill, R. W., Li, S., Karuturi, R. K. M., & George, J. (2020). CUP-AI-Dx: A tool for inferring cancer tissue of origin and molecular subtype using RNA gene-expression data and artificial intelligence. *EBioMedicine*, *61*, 103030. https://doi.org/10.1016/j.ebiom.2020.103030 | Data cannot be extracted |
| 195 | Zhu, X. H., Li, X. M., Zhang, W. L., Liao, M. M., Li, Y., Wang, F. F., Shang, B., Peng, L. G., Su, Y. J., You, Z. J., Shi, J. Y., Zhong, W. L., Liang, X. R., Liang, C. J., Liang, L., Liao, W. T., & Ding, Y. Q. (2021). *Zhonghua bing li xue za zhi = Chinese journal of pathology*, *50*(4), 333–338. https://doi.org/10.3760/cma.j.cn112151-20201013-00780 | Data cannot be extracted |
| 196 | Granata, V., Bicchierai, G., Fusco, R., Cozzi, D., Grazzini, G., Danti, G., De Muzio, F., Maggialetti, N., Smorchkova, O., D'Elia, M., Brunese, M. C., Grassi, R., Giacobbe, G., Bruno, F., Palumbo, P., Grassi, F., Brunese, L., Grassi, R., Miele, V., & Barile, A. (2021). Diagnostic protocols in oncology: workup and treatment planning. Part 2: Abbreviated MR protocol. European review for medical and pharmacological sciences, 25(21), 6499–6528. https://doi.org/10.26355/eurrev_202111_27094 | Protocol |
| 197 | Śniadecki, M., Jaworek, P., Chmielewska, Z., Poniewierza, P., Stasiak, M., Danielkiewicz, M., Stencelewski, D., Brzeziński, M., Boyke, Z. A., Wycinka, E., Sunil, M., Nguyen, M., Klasa-Mazurkiewicz, D., Koziełek, K., Rak, P., Wolny, Y., Liro, M., Guzik, P. W., Dobruch-Sobczak, K., & Wydra, D. (2023). Protocol of Breast Cancer Prevention Model with Addition of Breast Ultrasound to Routine Gynecological Visits as a Chance for an Early Diagnosis and Treatment in 25 to 49-Year-Old Polish Females. Diagnostics (Basel, Switzerland), 13(2), 227. https://doi.org/10.3390/diagnostics13020227 | Protocol |
